# Spike N354 glycosylation augments SARS-CoV-2 fitness for human adaptation through structural plasticity

**DOI:** 10.1093/nsr/nwae206

**Published:** 2024-06-14

**Authors:** Pan Liu, Can Yue, Bo Meng, Tianhe Xiao, Sijie Yang, Shuo Liu, Fanchong Jian, Qianhui Zhu, Yuanling Yu, Yanyan Ren, Peng Wang, Yixin Li, Jinyue Wang, Xin Mao, Fei Shao, Youchun Wang, Ravindra Kumar Gupta, Yunlong Cao, Xiangxi Wang

**Affiliations:** CAS Key Laboratory of Infection and Immunity, National Laboratory of Macromolecules, Institute of Biophysics, Chinese Academy of Sciences, Beijing 100101, China; University of Chinese Academy of Sciences, Beijing 100049, China; CAS Key Laboratory of Infection and Immunity, National Laboratory of Macromolecules, Institute of Biophysics, Chinese Academy of Sciences, Beijing 100101, China; Cambridge Institute of Therapeutic Immunology & Infectious Disease (CITIID), University of Cambridge, Cambridge CB2 0AW, UK; Biomedical Pioneering Innovation Center (BIOPIC), Peking University, Beijing 100080, China; Changping Laboratory, Beijing 102206, China; Joint Graduate Program of Peking-Tsinghua-NIBS, Academy for Advanced Interdisciplinary Studies, Peking University, Beijing 100871, China; Changping Laboratory, Beijing 102206, China; Joint Graduate Program of Peking-Tsinghua-NIBS, Academy for Advanced Interdisciplinary Studies, Peking University, Beijing 100871, China; Peking-Tsinghua Center for Life Sciences, Tsinghua University, Beijing 100084, China; Changping Laboratory, Beijing 102206, China; Chinese Academy of Medical Sciences & Peking Union Medical College, Beijing 100006, China; Biomedical Pioneering Innovation Center (BIOPIC), Peking University, Beijing 100080, China; Changping Laboratory, Beijing 102206, China; CAS Key Laboratory of Infection and Immunity, National Laboratory of Macromolecules, Institute of Biophysics, Chinese Academy of Sciences, Beijing 100101, China; University of Chinese Academy of Sciences, Beijing 100049, China; Changping Laboratory, Beijing 102206, China; CAS Key Laboratory of Infection and Immunity, National Laboratory of Macromolecules, Institute of Biophysics, Chinese Academy of Sciences, Beijing 100101, China; Changping Laboratory, Beijing 102206, China; CAS Key Laboratory of Infection and Immunity, National Laboratory of Macromolecules, Institute of Biophysics, Chinese Academy of Sciences, Beijing 100101, China; CAS Key Laboratory of Infection and Immunity, National Laboratory of Macromolecules, Institute of Biophysics, Chinese Academy of Sciences, Beijing 100101, China; CAS Key Laboratory of Infection and Immunity, National Laboratory of Macromolecules, Institute of Biophysics, Chinese Academy of Sciences, Beijing 100101, China; Changping Laboratory, Beijing 102206, China; Changping Laboratory, Beijing 102206, China; Cambridge Institute of Therapeutic Immunology & Infectious Disease (CITIID), University of Cambridge, Cambridge CB2 0AW, UK; Biomedical Pioneering Innovation Center (BIOPIC), Peking University, Beijing 100080, China; Changping Laboratory, Beijing 102206, China; Joint Graduate Program of Peking-Tsinghua-NIBS, Academy for Advanced Interdisciplinary Studies, Peking University, Beijing 100871, China; CAS Key Laboratory of Infection and Immunity, National Laboratory of Macromolecules, Institute of Biophysics, Chinese Academy of Sciences, Beijing 100101, China; University of Chinese Academy of Sciences, Beijing 100049, China; Changping Laboratory, Beijing 102206, China

**Keywords:** coronavirus glycosylation, viral fitness, adjustable infectivity, co-factor usage, viral evolution, conformational modulator

## Abstract

Selective pressures have given rise to a number of SARS-CoV-2 variants during the prolonged course of the COVID-19 pandemic. Recently evolved variants differ from ancestors in additional glycosylation within the spike protein receptor-binding domain (RBD). Details of how the acquisition of glycosylation impacts viral fitness and human adaptation are not clearly understood. Here, we dissected the role of N354-linked glycosylation, acquired by BA.2.86 sub-lineages, as a RBD conformational control element in attenuating viral infectivity. The reduced infectivity is recovered in the presence of heparin sulfate, which targets the ‘N354 pocket’ to ease restrictions of conformational transition resulting in a ‘RBD-up’ state, thereby conferring an adjustable infectivity. Furthermore, N354 glycosylation improved spike cleavage and cell–cell fusion, and in particular escaped one subset of ADCC antibodies. Together with reduced immunogenicity in hybrid immunity background, these indicate a single spike amino acid glycosylation event provides selective advantage in humans through multiple mechanisms.

## Teaser

Human coronaviruses (HCoVs) that cause seasonal colds achieve long-term adaptation in the human population through a high “glycan shield”. The recent emergence of the SARS-CoV-2 BA.2.86 lineage, with additional N354 glycosylation on the S protein, is an important event in the evolution of SARS-CoV-2 into a human seasonal coronavirus. N354 glycosylation makes the virus more susceptible to the upper respiratory tract, but weakens its ability to replicate in the lower respiratory tract, while reducing the immunogenicity of the virus, which is conducive to future reinfection by the virus. These results suggest that the event of increased glycosylation promotes the coexistence of SARS-CoV-2 with humans.

## INTRODUCTION

The ongoing coronavirus disease 2019 (COVID-19) pandemic caused by severe acute respiratory syndrome coronavirus-2 (SARS-CoV-2) has lasted for several years. A number of variants with improved fitness and immune evasion capabilities have been documented during the course of the pandemic [1]. The emergence and circulation of Omicron represents a significant antigenic distance shift in the evolution trajectory of SARS-CoV-2 because this variant has over 30 mutations in its spike (S). Subsequently, several Omicron descendants, such as BA.2, BA.5, BQ.1, and XBB, have caused multiple waves of infections globally. The successive selection of these sublineages is primarily driven by immune pressure exerted by neutralizing antibodies present in human sera as a result of mass vaccinations or natural infections or breakthrough infections [2,3]. However, immune evasion often comes at the cost of impairment in functionality and selection for antibody-escaping variants as well as accumulation of near-neutral mutations have led to suboptimal codon usage, thereby impacting functionality [4]. Upon boosting with updated (Omicron-based) vaccine or single Omicron infection, immune responses to Omicron variants have been shown to be attenuated owing to the ‘original antigenic sin’ (the propensity of the immune system to preferentially use immunological memory based on a previous infection when a second slightly different version of that foreign pathogen is encountered). However, repeated Omicron exposures override ancestral SARS-CoV-2 immune imprinting, yielding high neutralizing titers against Omicron variants, including XBB sublineages [3]. Given the extent of immunity raised by repeated Omicron exposures today, evolution of the virus by more nuanced human adaptation to overcome immune imprinting through strategies, such as increasing the glycan shield, might be already under way.

The dense glycan shield consisting of 22–23 *N*-glycosylation sequons per protomer is an essential feature of SARS-CoV-2 S architecture. The glycans have been shown to play intrinsic and extrinsic roles in protein folding, modulating conformational activation and immune evasion [5,6]. Pathogenesis and selective sweeps analysis reveal that the evolution of glycosylation sites in SARS-CoV-2 S is intertwined with adaptive mutations of the amino acid sequence for successful cross-species transmission [7]. For instance, loss of *N*-glycosylation at position 370 has been demonstrated to increase the receptor binding domain (RBD) in the up conformation, and thereby its exposure and accessibility for receptor recognition, improving viral infectivity in humans [8]. Distinct from roles played by N370 glycosylation, many other glycans simply form a sugary barrier that shields antigenic epitopes vulnerable to neutralizing antibodies and immunogenic epitopes capable of eliciting neutralizing antibodies. Glycan shield density analysis reveals a strong correlation that viruses historically classified as ‘evasion strong’ [9] had significantly elevated glycan shield densities [10]. Consequently, sites of glycosylation are often positively selected during viral evolution in a human host to increase glycan shield density. These assist the virus in evading the immune system, with impacts on infectivity [11]. Therefore, acquisition of extra glycans that presumably improves viral fitness and adaptation in humans might have occurred over the long course of the SARS-CoV-2 pandemic.

Phylogenetic analysis of sarbecoviruses based on their S sequences reveals four clades: clade 1a (e.g. SARS-CoV-1), clade 1b (e.g. SARS-CoV-2), clade 2 (e.g. Rf1), and clade 3 (e.g. BtKY72) [12], among which coronaviruses from different clades display distinct clade-specific sequence characteristics at key sites shown to play roles in modulating viral infectivity, antigenicity, and cross-species transmission (Fig. S1A). In contrast to SARS-CoV-1, which emerged in 2002, was under control in 2003 and disappeared in 2004, SARS-CoV-2 seems to coexist with humans. After a prolonged period of nearly-complete global dominance of XBB subvariants, substantially mutated lineages, designated BA.2.86 sublineages, have quickly spread worldwide, out-competing XBB (Fig. S1B) [13–15]. BA.2.86 sublineages contain more than 30 mutations in the S when compared to XBB or its parental BA.2, and some of these mutations have been rarely observed in previously circulating variants (Fig. S1C) [13–15]. The substitution P621S is a feature of SARS-CoV-1 variants and P681R is a fusion-enhancing modulator contained in Delta [16]. Both P621S and P681R have been selected in the BA.2.86 lineage (Figs S1A, S2A). The mutation K356T, predicted to acquire glycosylation at N354 due to the formation of a standard N-linked glycosylation site motif (NXT/S) occurred only in recently emerging SARS-CoV-2 variants, rather than in early SARS-CoV-2 variants and sarbecoviruses from other clades (Figs S1A and S2A). In addition, the new substitution of H245N in BA.2.86 yields one extra glycan at N245, further suggesting a gradual accumulation of a glycan shield. Coincidentally, a distinct footprint of positive selection located around a new non-synonymous change (A1067C; K356T) within the RBD was found through scanning over 180 000 SARS-CoV-2 genomes deposited from September 1, 2023 to January 1, 2024, indicating a selective sweep (Fig. S1D). Details of how the acquisition of glycosylation sites impacts the fitness of the virus are not clearly understood.

## RESULTS

### N354 glycosylation modulates RBD conformation

**Figure 1.**
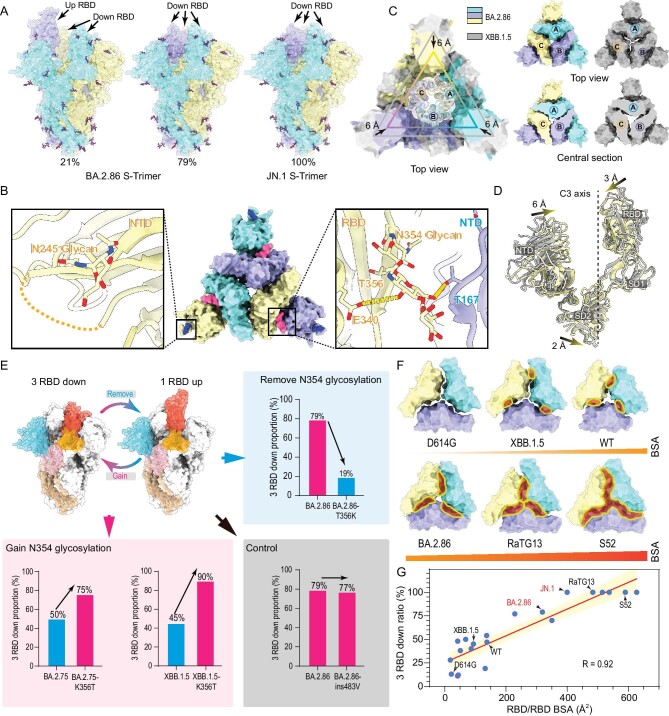
N354 glycosylation modulates RBD conformation. (A) Surface characterization of S-trimer of BA.2.86 and JN.1 in ‘up’ and ‘closed’ conformational states. The three subunits of S protein are colored in yellow, light blue, and purple, respectively, and the N-glycans are highlighted with sticks. (B) The glycosylation modifications at N245 and N354 in BA.2.86 sublineages are shown in detail. (C) RBD of the ‘closed’ conformation of BA.2.86 S-trimer superimposed with the XBB.1.5 S-trimer. Top view (top right) and center section (bottom right) show intersubunit contacts of BA.2.86 and XBB.1.5 S-trimers. (D) The conformational change details between BA.2.86 (yellow) and XBB.1.5 (gray) in S1 subunit. The shift distances and directions of NTD, RBD, and SD2 towards the 3-fold axis are labeled. (E) The role of N354 glycosylation in regulating changes in the ‘up’ and ‘down’ conformational ratio of the RBD in ‘Remove N354 glycosylation’, ‘Gain N354 glycosylation’ and ‘Control’ groups. In each group, the proportion of ‘RBD-down’ conformation are displayed with a bar chart. Blue and pink bars represent variants without and with N354 glycosylation. (F) The buried surface areas (BSA) between RBDs of D614G, XBB.1.5, WT, BA.2.86, RaTG13, and S52 are compared. (G) A correlation plot created between the contact area between RBD subunits and the ‘RBD-down’ rate.

To explore the putative acquisition of glycosylation at N245 and N354 in more recent variants, we determined the asymmetric cryo-EM reconstructions of the BA.2.86 and JN.1 S-trimer at pH 7.4, to mimic the physiological conditions at atomic resolution (Fig. 1A, Fig. S3A, S3B, and Table S1). In contrast to S-trimers from most variants ranging from WT, D614G through Alpha, Delta, Omicron to XBB and XBB.1.5, which sample the RBD-up conformation more frequently (>50%), BA.2.86 and JN.1 S-trimers dominantly adopt a closed state with all three RBDs in the down configuration (Fig. 1A), similar to structural observations of VAS5, a highly attenuated SARS-CoV-2 vaccine candidate [17]. In line with these structural observations, BA.2.86 was previously reported to have compromised infectivity and attenuated pathogenicity in animal models [18,19]. Compared to other variants, there are two additional glycosylation related modifications at N245 and N354 in BA.2.86 sublineages, among which N245 glycan lies at outermost region of each NTD around the triangular vertices of the S-trimer (Fig. 1B). Notably, the N354 glycan resides in a cleft formed by the NTD and RBD from two neighboring subunits, and establishes hydrogen bonds with T167 of NTD and with E340 of RBD, respectively, acting like a ‘bolt’ to lock the S-trimer in an ‘RBD-down’ state (Fig. 1B and C). This is akin to roles played by LA, a polyunsaturated fatty acid found in RBD in stabilizing the RBD-down state by locking the conformation of the S-trimer [20]. In line with this, the N354 glycosylation confers a more compact architecture in the region formed by the three NTDs and RBDs (Fig. 1C). When the closed S-trimers were superimposed with its counterparts from XBB.1.5, the NTD, RBD, and SD1 from BA.2.86 moved inward to the 3-fold axis with the shift distances of 6 Å, 3 Å, and 2 Å, respectively (Fig. 1D), forming a tight packing between NTD and RBD.

To further verify the role of N354 glycosylation in modulating RBD up/down disposition, four additional modified constructs, BA.2.86-T356K, predicted to remove glycosylation at N354, XBB.1.5-K356T and BA.2.75-K356T, predicted to acquire N354 glycan, together with BA.2.86-ins483V as a control, were characterized, and their structural features were compared with the ancestral strain (Fig. 1E and Fig. S3C). Indeed, various factors such as purification method, buffer conditions, and cryo-EM sample preparation may, to some extent, affect the RBD up/down ratio [21]. Thus, we performed all assays under the same condition by using three representative variants (BA.2.86, BA.2.75, and XBB.1.5) with strict controls (loss and gain of N354 glycan). We observed that N354 glycosylation dramatically increased the proportion of the ‘closed’ form from 19% to 79% in BA.2.86, 50% to 75% in BA.2.75, and 45% to 90% in XBB.1.5, making the ‘closed’ form the dominant population (Fig. 1E). However, the insertion of V483 had little effect on the modulation of the RBD conformation (Fig. 1E), suggesting the ‘closed’ and ‘open’ form transition was not due to the general effects of mutations in RBD but the specific presence of N354 glycosylation. Results of our studies together with previous studies on N-glycans at N165, N234, and N370 (not found in all SARS-CoV-2 variants) capable of participating in RBD up/down disposition [5,8], allows us to propose a detailed molecular basis for RBD conformation modulation, in which compact inter-subunit (S1/S1) arrangements relay a cascade of interactions mediated by specific N-glycans via tight connections with neighboring subunits or intrinsic packing modes, facilitating the RBD-down switch (Fig. 1F). To further decipher the relationship between inter-subunit contacts and ‘RBD-down’ rate, we systematically analyzed the S1/S1 or RBD/RBD or NTD-RBD/NTD-RBD interactions and calculated the ‘RBD-down’ rates of available SARS-CoV-2 S-trimer structures (*n* = 21), including ours in this study. We found that contact areas between RBDs determine the ‘RBD-down’ rate with a compelling correlation of 0.92 (Fig. 1F and Fig. S3D). Of note, RBD/RBD contact areas of over 400 Å^2^ drive the S-trimer to be in the closed state only, which is a common feature in animal derived sarbecoviruses, such as bat RaTG13, pangolin PCoV_GX, and BANAL-20–52. However, those sarbecoviruses are able to bind ACE2, but with decreased infectivity in human cells [22–24]. These results led us to hypothesise that the N354 glycan, nestled between the NTD and RBD interface, may function as a conformational control element for modulating infectivity.

### N354 glycosylation decreases infectivity irrespective of comparable hACE2 binding

**Figure 2.**
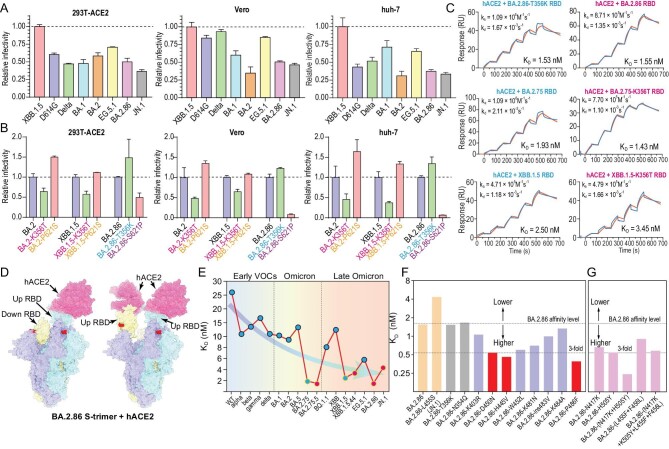
N354 glycosylation decreases infectivity, but not via compromising binding to hACE2. (A) Relative infectivity of XBB.1.5, D614G, Delta BA.1, BA.2, EG.5.1, BA.2.86, and JN.1. Vesicular stomatitis virus-based pseudoviruses were used to test the efficiency of infecting 293T-ACE2, Vero, and Huh-7 cells. Error bars represent the mean ± SD of three replicates. All raw data of infectivity are normalized by XBB.1.5. (B) Relative infectivity of BA.2, XBB.1.5, and BA.2.86 variants with mutations at positions 356 and 621, compared to their respective wild types, evaluated in 293T-ACE2, Vero, and Huh-7 cells. Error bars represent the mean ± SD of three replicates. (C) The impact of glycosylation at position 354 of BA.2.86, BA.2.75, and XBB.1.5 RBDs on the binding affinity to hACE2 assessed by SPR. (D) Surface characterization of two ‘up’ RBD conformations of BA.2.86 S-trimer binding to hACE2 determined by cryo-EM. The color scheme for three subunits of S are consistent with Fig. 1A and hACE2 is colored in pink. (E) Changes in affinity of binding hACE2 from early SARS-CoV-2 variants of concern (VOCs) and Omicron variants to late Omicron variants. (F) The effect of a single substitution on the binding affinity to hACE2 was assessed using SPR. Mutations that greatly enhance, moderately enhance, and decrease the affinity to hACE2 are indicated in red, light purple, and yellow, respectively. The cutoff value of greatly increasing affinity is set as a 3-fold change in K_D_ value relative to BA.2.86. (G) Evaluation of binding affinity to hACE2 of the variants with 1–2 mutations on the RBM of BA.2.86 by SPR. These variants are based on predictions of increased binding affinity to hACE2.

Given the fact that the presence of the N354-linked glycan favors the closed state in BA.2.86 sublineages, this presumably leads to a compromised infectivity and attenuated pathogenicity. To verify this, we first compared the infectivity of BA.2.86 and representative SARS-CoV-2 variants by using pseudotyped viruses in HEK293T cells overexpressing hACE2 (293T-ACE2) or TMPRSS2 (293T-TMPRSS2) or both hACE2 and TMPRSS2 (293T-ACE2-TMPRSS2) and in widely used cell lines, such as Vero, H1299, and Huh-7. Like Omicron variants, BA.2.86 can enter cells via endosomes as well as through TMPRSS2 but prefers ACE2-mediated infection (Fig. S4A) in concordance with authentic BA.2.86 infection results [25]. Overall BA.2.86 exhibited a partially decreased infectivity compared to most Omicron variants (Fig. 2A and Fig. S4A), which largely matches with recent studies [13–15], albeit with improved entry into lung cells rather than other cells relative to specific variants being observed as well [26,27]. These *in vitro* findings correlate to *in vivo* clinical observations that currently there are no reports of elevated disease severity associated with this variant [28]. Virus-host receptor engagement and membrane fusion directly affect viral infection efficiency. To further investigate if the RBD-dynamics modulator (N354 glycosylation) and putative fusion-related mutation (P621S) impact infectivity, we constructed BA.2/XBB.1.5/BA.2.86 derivatives that bear respective mutations and measured their infectivity in 293T-ACE2, Vero, and Huh-7 cells (Fig. 2B). As expected, acquisition of N354 glycosylation generated by the K356T mutation in BA.2 and XBB.1.5 decreased its infectivity, and loss of N354 glycosylation raised by the reverse mutation T356K in BA.2.86 increased its infectivity (Fig. 2B). Surprisingly, the substitution of P621S predicted to affect fusion activity in BA.2 and XBB.1.5 contributed to the increased infectivity; in turn the reverse substitution of S621P in BA.2.86 resulted in further decreased infectivity (Fig. 2B). Coincidentally, the N354 glycosylation (K356T mutation) first emerged in BA.2.75.5 and then in XBB.1.5.44, but they did not display growth advantages compared to the prevalent variants, presumably due to the dramatically reduced infectivity (Fig. S2A). S621P to a large extent compensated the decreased infectivity conferred by the N354 glycosylation.

We next sought to examine the possibility that the N354 glycosylation mediated impaired infectivity may be related to decreased binding affinity to hACE2. For this, three paired groups of six variants (BA.2.75/BA.2.75.5; XBB.1.5/XBB.1.5.44; BA.2.86/BA.2.86-T356K) were carefully selected due to the only difference being the presence of N354 glycosylation or not in their RBDs. Surface plasmon resonance (SPR) results demonstrated that RBDs with or without the N354 glycosylation showed comparable binding affinities to hACE2, indicating that the N354 glycosylation does not impact hACE2 binding (Fig. 2C). Moreover, the binding affinities of the BA.2.86 S-trimers with or without the N354 glycosylation for hACE2 yielded similar results (Fig. S4E), despite N354 glycosylation promoting the S-trimer ‘RBD-down’ state. To further structurally verify this, we determined the cryo-EM structure of the BA.2.86 S-trimer in complex with hACE2 (Fig. 2D, Fig. S4B, and Table S1). Like most complex structures, one or two copies of hACE2 are bound to the RBDs in the up configuration (Fig. 2D). Consistent with binding results, both the N354 glycan and T356 are located far away from the interface (Fig. S4C). However, we noted that variants with the N354 glycosylation exhibited very high affinities to hACE2, reflecting that tight binding might be required to further recover the compromised infectivity (Fig. 2E). To further explore the contribution on hACE2 tight binding exemplified by BA.2.86, we evaluated the individual substitution (including reverse mutation) of N354Q, T356K, K403R, D450N, H445V, W452L, L455S, K481N, ins483V, K484A, or P486F in BA.2.86 RBD on the hACE2 affinity. Surprisingly, all single mutations except for L455S identified in JN.1 displayed, to some extent, increased binding affinities and single reverse mutation of D450N, H445V, and P486F induced an ∼3-fold affinity increase (Fig. 2F). Furthermore, mutations identified in key variants were also evaluated, among which a reverse-mutated combination of N417K and H505Y, as well as a pair of Flip-mutations of L455F and F456L synergistically enhanced hACE2 binding (Fig. 2G). Structural comparisons revealed that the substitutions of N417K and H505Y established extra hydrogen bonds with D30 and E37 on ACE2, and mutations, including P486F, H445V, W452L and L455F-F456L augmented hydrophilic interactions of the microenvironment, increasing binding capabilities (Fig. S4D). These suggest that successful selection for acquisition of the N354 glycosylation possibly needs to be accompanied by tighter ACE2 binding together with the P621S substitution, co-manipulating the infectivity.

### Decreased infectivity by N354 glycosylation can be restored by HS

**Figure 3.**
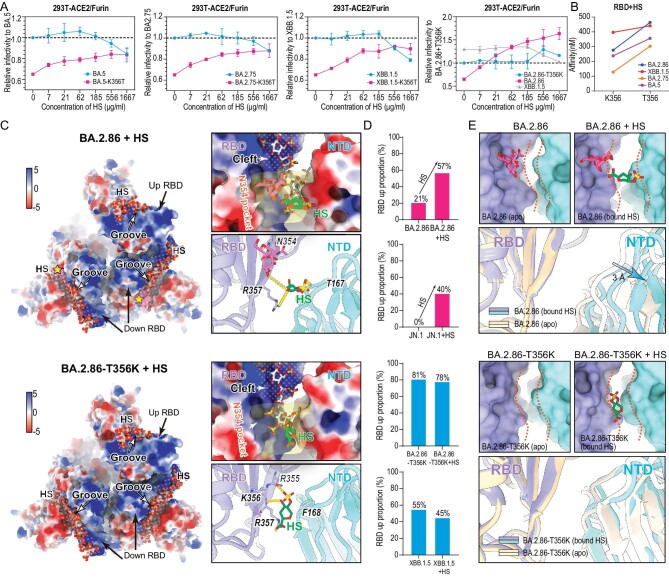
Mechanism of the ability of heparan sulfate recovering decreased infectivity by N354 glycosylation. (A) The infectivity of BA.5, BA.2.75, XBB.1.5, BA.2.86-T356K (blue) and their corresponding K356T (pink) mutant virus-like particles (SC2-VLPs) in 293T-ACE2/Furin cells with or without preincuation with increasing concentraion of HS. (B) Binding affinity of RBDs of BA.5, BA.2.75, XBB.1.5, BA.2.86-T356K and their corresponding K356T mutant to HS tested by SPR. (C) The cryo-EM structures of BA.2.86 and BA.2.86-T356K S-trimer bound to HS are shown in the upper and lower panels, respectively. In each panel's left corner, HS was docked to S-trimer by MOE. The binding grooves of HS are indicated by ‘dotted zones’ on the electrostatic surface of S-trimer. Pink surface of N354 glycosylation on BA.2.86 is highlighted by yellow stars. In the upper right corner, the explicit binding location of HS ‘N354 pocket’ in groove has been zoomed in and indicated by a light-yellow shadow. HS determined by cryo-EM and docked by MOE are colored in green and white, respectively. In the lower right corner, interface details of HS determined by cryo-EM with S-trimer are shown. Hydrogen bonds are displayed by yellow dashed lines. The unit of value for the color bar is kcal/(mol·e) at 298 K. (D) Influence of HS on the ‘RBD-up’ conformational proportion within the S-trimer of BA.2.86, JN.1, BA.2.86-T356K, and XBB.1.5. Both BA.2.86 and JN.1 are glycosylated at position N354, shown in red bars. BA.2.86-T356K and XBB.1.5, which lack glycosylation at position N354, are shown in blue bars. (E) Surface and cartoon representation of HS binding grooves consisting of a pair of spatially adjacent RBD (purple) and NTD (cyan) from different subunits of BA.2.86 (upper panel) and BA.2.86-T356K (lower panel). In each panel, apo state and HS-bound state are shown. Distance of HS binding grooves is indicated by orange dashed curves.

Viruses like influenza and coronavirus use glycans as entry factors. In particular, the initial interaction with host cells is mediated by glycans [29]. Growing evidence supports a role for negatively charged glycans, such as heparin sulfate (HS) as entry co-factors for SARS-CoV-2 [30]. More importantly, these entry co-factors and furin expression are specially more abundant in nasal epithelial cells and upper airway cells compared to those in the lungs [31]. Perhaps correlated with this, Omicron variants display a gradual increase in binding affinity to HS compared to early VOCs [32], presumably leading to a tropism alteration during SARS-CoV-2 evolution. Together with increased positive charges (Fig. S5A) and nearly all closed S-trimers mediated by the N354 glycosylation (Fig. 1A), these raise a possibility of altered entry factor usage in nasal epithelial and upper airway microenvironments. To mimic authentic virus infection at multiple steps of viral life cycles in nasal and upper airway tracks, we evaluated co-factor usage efficiency in representative variants via pre-treatment of virus-like particles (SC2-VLPs) [33] with various concentrations of free HS prior to infection by using 293T-ACE2-furin cells (Fig. 3A). We observed that free HS displayed a dose-dependent reduced infection of BA.5, BA.2.75, and XBB.1.5, consistent with previously reported inhibition in S binding and infection by authentic SARS-CoV-2 [30]. Surprisingly, HS treatment dramatically increased BA.2.86 infection, exceeding XBB.1.5 infectivity (Fig. 3A), which suggests that abundant HS and furin aided the recovery of the decreased infectivity for BA.2.86. To further decipher the underlying mechanism, we constructed BA.5-K356T, BA.2.75-K356T, XBB.1.5-K356T, and BA.2.86-T356K SC2-VLPs to gain or remove the N354 glycosylation, respectively, and compared the effect in HS treatment with their parental SC2-VLPs. Strikingly, all the N354 glycosylated SC2-VLPs exhibited a dose-dependent enhanced infectivity upon HS treatment, reaching up to the infectivity level for their parental variants and loss of the N354 glycosylation largely eliminated the HS-induced enhanced infectivity in BA.2.86 (Fig. 3A), indicative of differential usage of HS as a cofactor for modulating infectivity of the N354 glycosylated variants in furin/hACE2 enriched microenvironments. This observation has recently been reflected by experimental observations of potent infections for BA.2.86 in nasal epithelial cells [34]. In line with these results, the N354 glycosylation partially impaired binding of HS to RBDs (Fig. 3B). Intriguingly, HS-mediated enhancements of infectivity for the N354 glycosylated variants became marginal by using pseudo-typed viruses in 293T-ACE2 cells, while HS dose-dependent inhibitions of infectivity for variants without the N354 glycosylation were still straightforward (Fig. S5B). The possible reason for differences yielded from two systems might lie in excessive redundancy of spikes decorated on VSV-based pseudoviruses, in which limited numbers of the ‘open’ spikes can initialize a successful infection even though the majority are in the closed state, largely diluting roles played by HS in modulating infectivity of the N354 glycosylated variants via promoting the RBD-up transition. Collectively, these revealed that HS and furin enriched microenvironments might offset the impaired infectivity caused by the N354 glycosylation and even possibly support the shift in tropism towards HS-abundant cells.

To understand SARS-CoV-2 engagement of the HS cofactor and how the N354 glycan alters HS usage at the molecular level, we determined cryo-EM structures of XBB.1.5, BA.2.86, JN.1, and BA.2.86-T356K in complex with HS at 3.2–3.8 Å (Fig. 3C and Fig. S5C). Interestingly, incubation with HS led to marked conformational alteration, yielding substantially increased ‘RBD-up’ state in the N354 glycosylated S-trimers, but had limited impact in RBD conformation modulation for S-trimers without the N354 glycosylation (Fig. 3D). Due to structural heterogeneity and flexibility of HS, only the density for part of the HS basic unit, IdoA (2S) (2-O-sulfo-α-L-iduronic acid), is clear, allowing identification of the location of major binding sites and interactions (Fig. 3C). In contrast to binding of sialoglycan to the domain A (corresponding to the NTD in SARS-CoVs) in HKU1 and MERS-CoV [35,36], HS mainly targets a semi-open, shallow, elongated cavity composed of a number of positively charged residues on RBD, downstream within a deep groove, named the N354 pocket, constructed by the residues N354, R355, K/T356, and R357 from RBD and T157 and F168 from neighboring NTD, is occupied by the HS fragment (Fig. 3C). The HS fragment is poised to possibly interact with R355 and R357 through hydrogen bonds and a salt bridge, meanwhile residues K356, N354, R346, and R466 might contribute to further coordinate the oligosaccharide (Fig. 3C). Notably, the absence of the N354 glycan in the immediate vicinity of the binding groove probably facilitates unobstructed engagement of HS, in line with the observed affinity; however, the presence of the N354 glycan together with the bound HS widens the binding groove by 3 Å, pushing the neighboring NTD outwards and thereby conferring a relatively relaxed upper arrangement (Fig. 3E). The high proportion of the ‘3-RBD-down’ state led by the N354 glycan mediated compact upper architecture could be partially converted to the ‘RBD-up’ state upon HS binding, which explained the experimental observation that HS treatment increased infectivity for the N354 glycosylated variants.

### N354 glycosylation affects S cleavage and fusogenicity

**Figure 4.**
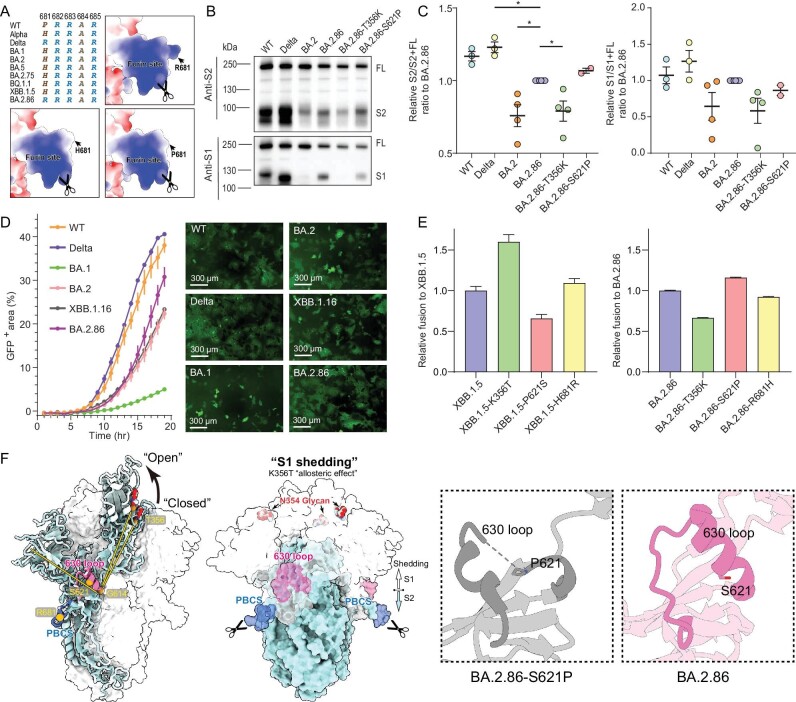
S cleavage and fusogenicity of BA.2.86. (A) Sequence alignment and modeled charge surface representation of PBCS consisting of P681, H681, and R681. S cleavage efficiency evaluated by Western blotting (B) and image grayscale analysis (C) for WT, Delta, BA.2, BA.2.86, BA.2.86-T356K, and BA.2.86-S621P. (D) Fusogenicity among WT, Delta, BA.1, BA.2, XBB.1.16, and BA.2.86 by using a split GFP system. (E) Fusogenicity of spikes bearing K356T, P621S, and H681R mutation on XBB.1.5 relative to XBB.1.5 (left) and spikes bearing T356K, S621P, and R681H mutation on BA.2.86 relative to BA.2.86 (right). (F) The location of N354 glycan (red), 630 loop (pink), and PBCS (blue) on S-trimer is shown on the left. T356, G614, S621, and R681 are displayed as orange spheres. Pattern diagram of K356T increasing S1 shedding by allosteric effect is shown in the middle. Cartoon representations of 630 loop of BA.2.86-S621P and BA.2.86 are zoomed in on the right.

We next sought to examine the possibility that the impaired infectivity caused by the N354 glycosylation in some cells might be related to differential S cleavage. For instance, Delta, which is known to show higher infectivity, is associated with a highly cleaved S protein and more efficient TMPRSS2 usage for entry [37]. Furin cleavage dependent on the polybasic cleavage site (PBCS) between S1 and S2 is a key step in regulating virus infectivity and fusion activity [37–39]. Alterations at P681 in PBCS have been observed in multiple SARS-CoV-2 lineages, H681 in Alpha and most Omicron variants; R681 in Delta and BA.2.86 (Fig. 4A). To evaluate the cleavage efficiency, we first tested the cells used for WT, BA.2, and BA.2.86 pseudoviruses production by western blot analysis. We found substantially improved cleavage in BA.2.86 compared with BA.2 as evidenced by the ratio of S1/S2 to full-length S, despite being slightly lower than that in WT and Delta (Fig. 4B and C), suggesting that mutation at P681 contributes non-exclusively to S cleavage. To further investigate putative contribution on enhanced cleavage, we also evaluated S cleavage in BA.2.86-T356K and BA.2.86-S621P. Interestingly, the loss of N354 glycosylation through T356K mutation decreased cleavage efficiency and the reversion of S621P moderately increased S cleavage (Fig. 4C), indicative of BA.2.86 S-trimers being more likely in a postfusion conformation under furin enriched microenvironments. Given our data showing inefficient TMPRSS2 usage for BA.2.86 sublineages, N354 glycosylation appears to negatively correlate between cleavage efficiency and infectivity (Fig. 2B and Fig. 4C).

The ability of SARS-CoV-2 S to induce cell–cell fusion, providing an additional route for viral dissemination and promoting immune evasion, correlates with the PBCS, S cleavage efficiency, and the usage of TMPRSS2 [37]. Given the requirement of TMPRSS2 and S cleavage for optimal cell–cell fusion, Delta displayed the highest fusion activity; on the contrary BA.1 had quite low fusogenicity [38,39]. We hypothesized, based on the increased cleavage efficiency, that the fusion efficiency is altered in BA.2.86 in comparison to BA.2. To examine this, we used a split GFP system [37] to monitor cell–cell fusion in real time. We observed that BA.2.86 showed an increment in cell–cell fusion compared to BA.2, but was still demonstrably lower than WT and Delta (Fig. 4D). Fusion inhibitors like EK1 showed very potent fusion inhibitory activity against BA.2.86-S-mediated fusion [40]. The efficiency in fusion is reversely correlated with the stabilities of S-trimers (Fig. S6A), which can be explained by the fact that structural transitions from the prefusion to postfusion stage involve a series of conformational changes between domains and subunits, a prerequisite for viral fusion. Structural comparisons with BA.1 revealed reduced interactions between domains, including NTD-RBD, RBD-SD1/SD1-S2, and S2-S2 in BA.2.86, structurally explaining compromised stability (Fig. S6B). Not surprisingly, either the loss of the N354 glycan or substitution R681P/H in BA.2.86 substantially reduced the cell–cell fusion activity; on the contrary acquisition of the N354 glycan or the mutation H681R based on XBB.1.5 contributed to increased fusion activity (Fig. 4E). The improved S processing and fusion might be related to the structural observation through an allosteric mechanism that the N354 glycan tightly cements the NTD and RBD from adjacent subunits together presumably aiding in S1 shedding, a pre-requisite step for subsequent fusogenicity. As expected, the single mutation S621P based on BA.2.86 improved the fusion activity and the mutation P621S in XBB.1.5 dramatically decreased its fusion efficiency (Fig. 4E). In line with functional observations, the mutation P621S facilitates formation of an α-helix in the 630 loop, a key modulator for fusion [1,41], that would be adopted as a partially disordered loop in P621 variants, to some extent structurally impeding structural rearrangements for subsequent fusion (Fig. 4F). These data indicate that the N354 glycosylation coupled with P621S alters multiple virological characteristics, in which cell–cell fusion activity renders the N354 glycosylated variants difficult to be neutralized by antibodies.

### K356T coupled N354 glycosylation specially escapes a subset of ADCC antibodies

**Figure 5.**
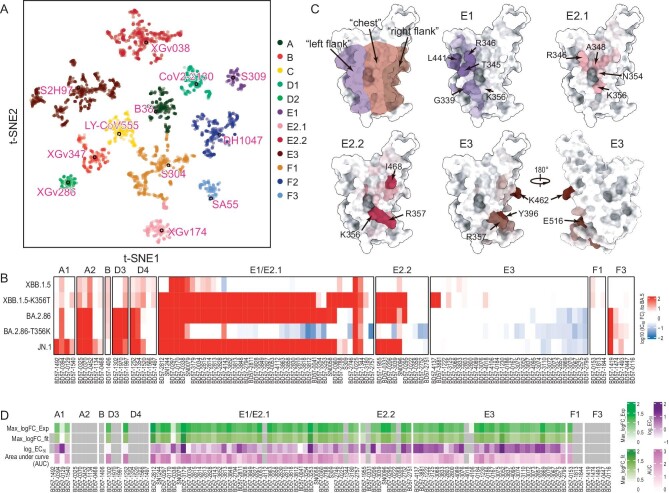
K356T coupled N354 glycosylation specially escapes a subset of ADCC antibodies. (A) t-SNE and unsupervised clustering of antibodies that bind SARS-CoV-2 RBD. Twelve epitope groups were identified from the DMS dataset (3051 antibodies). (B) Heatmap of neutralizing activity against XBB.1.5, XBB.1.5-K356T, BA.2.86, BA.2.86-T356K, and JN.1 of representative antibodies from 10 epitope groups, relative to BA.5. (C) Mapping of escape scores for antibodies from epitope group E1 (‘left flank’), E2.1 (‘chest’), E2.2 (‘chest’), and E3 (‘right flank’) on SARS-CoV-2 RBD (PDB: 6M0J). (D) Heatmap of ADCC effect of RBD antibodies. Four types of color bars represent the base 10 logarithm of the maximum of experiment curve, the base 10 logarithm of the maximum of the fitting curve by four parameters fitting, the base 10 logarithm of EC50 and area under curve from Fig. S7. The antibodies with ‘grey bar’ representations were not selected to perform ADCC assays. These antibodies cannot bind to the WT RBD, but the surface antigens of the target cell for ADCC assays are from SARS-CoV-2 WT variant.

Major selective pressures for previous VOCs, such as Delta, BA.2, BA.5, and XBB causing waves of infections globally, came from specific classes of antibodies driving immune evasion [2]. Compared to FLip and other XBB variants, BA.2.86 did not show substantial humoral immune escape, while JN.1 with one additional mutation (L455S) on BA.2.86 became more immune-evasive due to extensive resistance across three types of antibodies [42]. Previously, we determined the escape mutation profiles and epitope distribution of a total of 3051 antibodies isolated from vaccinated or breakthrough infection (BTI) individuals by deep mutational scanning (DMS), which were classified into 12 subgroups (Fig. 5A). Immune evasion pattern assays revealed that BA.2.86 sublineages specifically escaped A2, D3, part D4, and many E antibodies when compared to XBB.1.5 (Fig. 5B). Strikingly, acquisition of the N354 glycosylation by the K356T substitution largely inactivated group E1, E2.1, and E2.2 antibodies, although these antibodies displayed relatively low but broad neutralizing activities (Fig. 5B and Fig. S7A). Class E antibodies from E1 to E3 target epitopes on the RBD ranging from left flank through chest to right flank, and most E1, E2.1, and E2.2 antibodies extensively associate with K356 and N354, which has been validated by complex structures, including S309 (E1) (Fig. 5C and Fig. S7B). The mutation K356T could decrease charge/hydrophilic interactions and the N354 glycan fatally induced steric clashes, disabling the binding of most E1, E2.1, and E2.2 antibodies (Fig. S7B). Fc-dependent effector mechanisms, e.g. antibody-dependent cell cytotoxicity (ADCC) mediated by natural killer cells, could facilitate viral clearance from infectious individuals. Remarkably, we observed efficient E antibodies-mediated ADCC of SARS-CoV-2 S-transfected cells (Fig. 5D and Fig. S7C), revealing that the N354 glycosylated variants coupled with K356T specially escapes one subset of ADCC antibodies. Of note, there is one limitation that antibodies from other classes possibly possessing ADCC activities, were not tested here due to no cross-reactivity to WT spikes in our ADCC system. Together with improved cell–cell fusion, these possibly make the N354 glycosylated variants difficult to be cleared from individuals infected with virus.

### N354 glycosylation reduces immunogenicity in a hybrid immunity background

**Figure 6.**
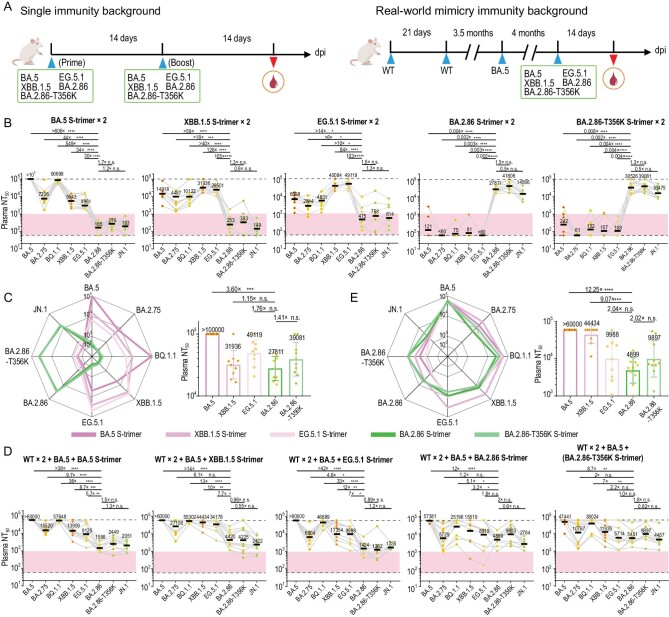
N354 glycosylation reduces immunogenicity in a hybrid immunity background. (A) Two cohorts of mice evaluating the immunogenicity of various SARS-CoV-2 variants. One cohort consisted of non-immunized BALB/c mice that received two doses of spike proteins (BA.5, XBB.1.5, EG.5.1, BA.2.86, BA.2.86-T356K), with a 14-day interval between each dose. The other cohort mimicked a real-world immunity background, where BALB/c mice were immunized with an inactivated vaccine (two doses of WT + one dose of BA.5) in addition to a single dose of spike protein (BA.5, XBB.1.5, EG.5.1, BA.2.86, BA.2.86-T356K). Blood samples were collected 14 days after immunization. The 50% neutralizing titer (NT50s) against Omicron variants (BA.5, XBB.1.5, EG.5.1, BA.2.86, BA.2.86-T356K) in plasma from a non-immunized BALB/c mice background (B) and from BALB/c mice simulating a real-world immune background (D). The *p*-values were calculated via a two-tailed Wilcoxon signed-rank test for paired samples. Radar plots of the spectrum of neutralization and bar charts of the immunogenicity of the five types of immunogens from a single immunity background (C) and a real-world mimicry immunity background (E).

In addition to immune escape, viruses generally evolve to acquire new glycosylation sites on the protein surface, a natural phenomenon of glycan shielding, which alters their glycoprotein immunogenicity [43]. To investigate if the N354 glycosylation may affect its immunogenicity, we first assessed humoral immune responses in naive (non-immunized) BALB/c mice following two-dose primary series immunization with variant S proteins (Fig. 6A). All S proteins contained six proline substitutions (S6P) and mutations in the PBCS to stabilize them in the prefusion conformation [9]. Groups of mice (*n* = 10 per group) were inoculated intramuscularly with 10 μg of variant S proteins, a widely used dosage for immunogenic evaluations in mice [3,44], including BA.5, XBB.1.5, EG.5.1, BA.2.86, and BA.2.86-T356K, on days 0 and 14, and sera were collected at day 28 (2 weeks after the second dose). Administration of BA.5, XBB.1.5, and EG.5.1 S proteins exhibited very low serum 50% neutralizing titers (NT_50_) against BA.2.86, BA.2.86-T356K, and JN.1 (using vesicular stomatitis virus-based pseudovirus), meanwhile immunization of BA.2.86 and BA.2.86-T356K resulted in quite limited neutralizing titers against Omicron sublineages, suggesting a large antigenic distance between Omicron and BA.2.86 from single immunity background analysis (Fig. 6B and C). Notably, the N354 glycosylation decreased BA.2.86 immunogenicity by ∼40% in comparison with BA.2.86-T356K, rendering BA.2.86 a relatively lower immunogenicity among SARS-CoV-2 variants (Fig. 6C).

To further evaluate the effects of the N354 glycosylation in SARS-CoV-2 immune imprinting induced by breakthrough infections, we modeled a real-world mimicry immunity background in mice. To accomplish this, two doses of 0.3 μg CoronaVac (1/10 human dose, an inactivated vaccine derived from WT) were used as primary immunization, then one dose of 0.3 μg inactivated BA.5 vaccine was administrated at 3.5 months after the second dose to mimic BA.5 BTI, and one dose of 10 μg variant S protein at 4 months after the third dose was used to mimic BTI + reinfection (Fig. 6A). Compared to single immunity background, single-dose administration of Omicron BA.5, XBB.1.5, and EG.5.1 S proteins under a hybrid immunity background displayed ∼5–20-fold improved cross neutralization against BA.2.86 sublineages and single-dose immunization of BA.2.86 and BA.2.86-T356K could produce ∼50–200-fold increased neutralizing titers against Omicron subvariants (Fig. 6D and E), suggesting that the existence of hybrid immune imprinting facilitates cross-reactive B cell recall and shortens antigenic distance. As ongoing evolution, an intrinsic trend in gradually decreased immunogenicity for variant S proteins was observed and acquisition of the N354 glycan further induced ∼2-fold reduction in the immunogenicity under a hybrid immunity background, consequently conferring alleviated immune imprinting (Fig. 6E). Nonetheless, a one-dose booster of BA.2.86, in particular BA.2.86-T356K, under real-world mimicry of immunity background could elicit high levels of neutralizing antibodies against BA.2.86 sublineages, including the currently prevalent JN.1 (Fig. 6E), revealing that immune responses can be fine-turned to the BA.2.86 sublineages by boosting with a tweaked (BA.2.86-based) vaccine. These indicate an altered evolution trajectory towards more sophisticated adaptation in humans through acquisition of the N354 glycan.

## DISCUSSION

A selectively favorable mutation spreading all or part of the way through the population generally causes a decrease in the level of sequence variability at nearby genomic sites [45], which can be manifested as a selective sweep signature. By using OmegaPlus and RAiSD, we mapped putative sweep regions in 184 224 SARS-CoV-2 genomes deposited in the past 4 months (from September 1, 2023 to January 1, 2024) from the GISAID EpiCoV database (see Materials and Methods). Four similar selective sweep regions were detected in the S from both datasets regardless of whether wild type or BA.2 or BA.5 or XBB was used as a reference (Fig. S1D). Two non-synonymous changes (A1067C and A1114G) within the codons for residues 356 (K→T) and 372 (T→A) of RBD were centrally located in one of the sweep regions, leading to acquisition and loss of N354 and N370 glycosylation, respectively (Fig. S1D). Loss of the N370 glycosylation has been shown to be an important evolutionary event for SARS-CoV-2 emergence from animal reservoirs and the enhanced human-to-human transmission during the early stages of the pandemic [7,8]. Our findings, to some extent, suggest that the N354 glycosylation acquired by variants during the course of the prolonged SARS-CoV-2 pandemic likely confers selective advantage for optimal adaptation in humans through a shift in tropism with adjustable infectivity, reduced immunogenicity, and elimination-escaped immune evasion.

The conformational dynamics of RBD, and modulation thereof, would render sarbecoviruses cunning to balance host cell attachment and immune escape. The transition to the ‘up’ state exposures of RBD for the binding to hACE2 is also a prerequisite for S-mediated viral fusion, directly correlating with infectivity. Thus, S proteins from most circulating SARS-CoV-2 variants have been observed in the RBD-up state with a reasonable proportion (>50%). Remarkably, however, recently prevalent BA.2.86 sublineages dominate their S protein in the RBD-down state up to 100% for JN.1 due to acquisition of the N354 glycosylation, shielding RBD from neutralizing antibodies and preventing RBD-hACE2 engagement. Surprisingly, the decreased infectivity could be recovered by altered binding mode of HS co-factor to promote the RBD-up conformational transition, apparently through an allosteric mechanism, conferring an adjustable infectivity and a shift in tropism towards HS-abundant cells.

During the process of viral evolution, viruses develop different glycosylation modifications, yielding appreciable impacts on survival, transmissibility, and fitness. In general, the majority of N-glycan adding mutants show decreased infectivity and transmission efficiency [46], in turn, immune-shielding glycans are beneficial for immune evasion, which reflects a sophisticated and balanced evolution strategy for N-glycan site accumulation. Further evidence for this has been documented in the viral evolution of Influenza A with additional N-glycan sites every 5–7 years [47]. Whether the limited glycan shield density observed on SARS-CoV-1, SARS-CoV-2, and Middle East syndrome coronaviruses (MERS) is correlated to the zoonosis of the pathogens is unknown. Notably, among *betacoronavirus* genus, seasonal human coronaviruses HKU1 and OC43 have long co-existed with humans and possess 26–31 N-glycan sites per S monomer, versus 22–23 N-linked glycan sequons in SARS- and MERS-CoVs (Fig. S2B). Remarkably, N-glycan sites on OC43 S were accumulated in the past 60 years with ∼2 N-glycan sites added every 20 years (Fig. S2C). A marginal trend in the relationship between N-glycan sites and prevalent time in humans was also observed in HKU1 presumably due to its first isolation and identification in 2004. It's tempting to speculate that adequate prevalent time might be required to monitor the glycan shield accumulation or HKU1 evolution to enter a relatively mature stage, bearing ∼30 and 5 N-glycan sites in S monomer and RBD, respectively (Fig. S2C). Even so, N-glycan modifications of coronavirus S proteins do not constitute a *bona fide* and effective shield, when compared to the glycan density of other viruses such as HIV, influenza, and Lassa, which may be reflected by overall structure, sparsity, oligomannose abundance, and immune evasion [48]. Although it's difficult to directly compare viruses in terms of immunogenic responses, SARS-CoVs readily elicit robust neutralizing antibodies that target S proteins following infection or immunization [49,50]. In contrast, the effective glycan shield of HIV hinders the production of sufficient immune responses and broadly neutralizing antibodies [51]. We speculated that the high plasticity of SARS-CoV-2 spike RBD may limit the accumulation of glycans on itself. The biological importance of the N354 glycosylation in modulation of SARS-CoV-2 immunogenicity and immune responses may provide implications in coronavirus vaccine research.

### Limitations of the study

Evaluation of virus infectivity *in vitro* by cell lines may not completely reflect the true infection efficiency of the virus *in vivo*. Due to limitations of Biosafety level 3 laboratories and related materials, studies on viral tropism and infections in primary cells/organoids are interesting, but they are beyond the scope of the present study. The evaluation of antibody escape also faces the same situation. Although the pseudovirus infection assay is classic and widely-used, immunological escape is a complicated process *in vivo*, requiring BSL3 laboratories and ideal animal models. Additionally, the immune background against SARS-CoV-2 for the population is very complex. While mouse-based animal models cannot reflect the human immune imprinting situation, it is challenging to perform related assays in humans due to the heterogenicity of the hybrid immunity from immunisation background and or breackthrough infections.

## Supplementary Material

nwae206_Supplemental_File
