# Supplementary material for: Spike N354 glycosylation augments SARS-CoV-2 fitness for human adaptation through structural plasticity
Source: Natl Sci Rev. 2024 Jun 14;11(7):nwae206. doi: 10.1093/nsr/nwae206 (PMC11282955; doi:10.1093/nsr/nwae206)
Supplement: nwae206_Supplemental_File [file nwae206_supplemental_file.docx]

Supplemental Information for

**Spike N354 glycosylation augments SARS-CoV-2 fitness for human adaptation through structural plasticity**

**Authors:** Pan Liu^1,2,†^, Can Yue^1,†^, Bo Meng^3,†^, Tianhe Xiao^4,5,6,†^, Sijie Yang^5,6,7,†^, Shuo Liu^5,8,†^, Fanchong Jian^4,5^, Qianhui Zhu^1,2^, Yuanling Yu^5^, Yanyan Ren^1^, Peng Wang^5^, Yixin Li^1^, Jinyue Wang^1^, Xin Mao^1^, Fei Shao^5^, Youchun Wang^5^*, Ravindra Kumar Gupta^3^*, Yunlong Cao^4,5,6^*, and Xiangxi Wang^1,2,5,^*

**This PDF file includes:**

Materials and Methods

Figs. S1 to S8

Table S1

**Materials and Methods**

**Putative Selective Sweep Region Detection**

Genomic scans for selective sweeps were performed by two programs. The one is OmegaPlus v3.0.3 [1] and the other is RAiSD v2.9 [2]. A total of 184,224 SARS-CoV-2 spike protein sequences were retrieved from the GISAID EpiCov database (https://www.gisaid.org/) from Sep.01,2023 to Dec.31,2023. Sequence reads were aligned to SARS-CoV-2 WT (NCBI Reference Sequence/NC_045512.2), BA2 (BA.2_hCoV-19/France/IDF-IPP08725/2022|EPI_ISL_10071318), BA5 (BA.5_hCoV-19/South_Africa/NCV1255/2022|EPI_ISL_12587877), XBB (XBB_hCoV-19/USA/NY-NYULH8854/2022|EPI_ISL_15427610) reference using Minimap2 [3]. In order to improve accuracy and filter out low quality sequences, sequences with aligned lengths less than 3,200 were excluded from the analysis, leaving 163,500 sequences for alignment using MAFFT [4]. OmegaPlus was performed with the following parameters: SARS-CoV-2 spike sequence was divided into 1,000 bins (-grid 1,000). The calculation of linkage disequilibrium values between SNPs utilized a defined window range, specifically set at 20bp as the minimum and 200bp as the maximum (-minwin 20 -maxwin 200). RAiSD was performed with the following parameters: The grid size to specify the total number of evaluation points was set to be 1,000 (-G 1,000); Missing data imputation was enabled (-M 1; per SNP). The sliding window size was set to be 200bp (-w200). Combination statistics of both programs, the top 20% intersecting regions detected were selected as the candidate sweep reigons (-COT 0.2).

**Protein expression and purification**

The full-length sequence information of Spike (S) from BA.2.86 was obtained from the NCBI (GenBank: WMV03218.1). The BA.2.86 spike and RBD protein genes were obtained by using the BA.2.75 gene as a template and performing overlapping PCR. The JN.1, BA.2.86-T356K, BA.2.86-ins483V Spike and JN.1, BA.2.86-T356K, BA.2.86-N354Q, BA.2.86-K403R, BA.2.86-D450N, BA.2.86-H445V, BA.2.86-W452L, BA.2.86-K481N, BA.2.86-ins483V, BA.2.86-K484A, BA.2.86-P486F, BA.2.86-N417K, BA.2.86-H505Y, BA.2.86-(N417K+H505Y), BA.2.86-(L455F+F456L), BA.2.86-(N417K+H505Y+L455F+F456L) RBD protein genes were obtained by using the BA.2.86 Spike and RBD genes as a template and performing overlapping PCR. The BA.2.75-K356T Spike and RBD protein genes were obtained by using the BA.2.75 gene as a template and performing overlapping PCR. The XBB.1.5-K356T Spike and RBD protein genes were obtained by using the XBB.1.5 gene as a template and performing overlapping PCR. To improve protein expression and stabilize the trimeric conformation, proline substitution was performed at residues 817, 892, 899, 942, 986, and 987 in all Spike gene constructs. And all spikes were modified to incorporate 2A mutations (R683A and R685A). Additionally, the C-terminus of the constructs was modified by adding the T4 fibritin folding domain. To facilitate protein purification, His or Strep II tags were attached at the C-terminus of all gene constructs. The spike and RBD proteins were obtained using a eukaryotic expression system. Plasmids containing the target protein were transiently transfected into suspended HEK293F cells at a density of 2×10^6^ cells/ml. The transfection efficiency was 80%~90%. After that, HEK293F cells were cultured at 37°C in a constant-temperature shaker with 8% CO_2_ for 72 hours. After collecting the cell supernatant, preliminary purification was performed using Ni-NTA or affinity StrepTactin resin chromatography. The proteins were further purified using Superdex 200 10/300GL (Cytiva) or Superose 6 10/300 (Cytiva) in phosphate-buffered saline (PBS) at pH 7.4 to obtain high-purity proteins.

**Surface Plasmon Resonance**

Surface plasmon resonance (SPR) was utilized for quantifying the binding affinity between the antigen and receptor, as well as the antigen and heparan sulfate (HS). In investigating interactions between the receptor and antigen, human ACE2 (hACE2) was immobilized as the stationary phase with the method of amine coupling reaction, while the SARS-CoV-2 RBDs acted as the mobile phase. For evaluating the affinity between HS and RBDs, the RBDs were immobilized as the stationary phase, and HS molecules were used as the mobile phase. We didn’t conduct regeneration processes. These experiments were conducted at a temperature of 25°C, employing the Biacore8K biosensor on the S series CM5 chip (Cytiva) for data detection and recording. The raw data curves were analyzed and fitted using the Biacore 8K evaluation software (GE Healthcare) employing a 1:1 binding model.

**Determination of Spike stability**

The stability of spike protein trimers of WT, Delta, BA.1, BA.2, BA.2.86, and XBB.1.16 at neutral pH (pH = 7.4) was evaluated using the ThermoFluor Assay. 5 μg sample of the spike protein was added to a 25 μl reaction system containing a final concentration of 1× SYPRO Orange dye (Invitrogen, USA) as a fluorescence probe. The protein was heated from 25°C to 99°C at a rate of 1°C/min using the QuantStudio™ 6 Flex Real-Time PCR System instrument (Applied Biosystems, USA), and changes in fluorescence signals were recorded during this temperature gradient. Data analysis and curve plotting were performed using GraphPad Prism 9.4.0 (GraphPad Software Inc.).

**Cryo-EM sample preparation and data collection** **and model building**

Purified Spike trimer protein samples from SARS-CoV-2 variants BA.2.86, JN.1, BA.2.86-T356K, BA.2.86-ins483V, BA.2.75-K356T, and XBB.1.5-K356T were diluted to a concentration of 1.0 mg/mL in PBS buffer, pH 7.4. Similarly, to prepare the spike/hACE2 complex sample, the BA.2.86 Spike protein was mixed with hACE2 at a molar ratio of 1:1.2, and the Spike/HS complex were mixed at a molar ratio of 1:1000, while maintaining a constant concentration of 1.0 mg/mL for the Spike. The support films were previously subjected to glow discharge or plasma cleaning for 20 seconds at a power of 25W, in the atmosphere of mixture of H_2_ and O_2_ gas flow*.* After that, 3 μl of the sample was pipetted onto pre-treated porous carbon-coated gold grid (C-flat, 300 mesh, 1.2/1.3, Protochips Inc.). The Vitrobot (FEI) was operated in a no-force mode to blot the sample for 6 seconds under 100% relative humidity and room temperature conditions. Subsequently, the sample was rapidly plunge frozen into liquid ethane.

Cryo-EM datasets were collected using a 200 kV FEI Krios ARCTICA or 300 kV FEI Titan microscope (Thermo Fisher) equipped with K2, K3, or Falcon 4 detectors. Movies were recorded with 32 frames at an exposure time of 0.2 seconds per frame, resulting in a total dose of 60 e ^–^ Å ^-2^. The automated single-particle data collection using SerialEM resulted in a final pixel size of 1 Å, 1.036 Å, 1.04 Å or 1.07 Å.

Data processing was performed using cryoSPARC (v4.3.0) and Relion (v3.0.8). The data underwent several steps including Motion Correction, CTF Estimation, Create Templates, Template Picker, Extract from Micrographs, 2D classification, 2D selection for Ab-initio Reconstruction, and subsequent Homogeneous Refinement. To enhance the density around the RBD/RBD-ACE2 region, local refinement was conducted using UCSF Chimera (v1.13.1) and CryoSPARC (v3.2.1). Structural modeling and refinement were performed using WinCoot (v0.9.8.1) and Phenix (v1.20.1). Figures were generated using UCSF ChimeraX (v1.6.1).

**Molecular Docking**

Electrostatic potential maps of the BA.2.86 S-trimer and BA.2.86-T356K S-trimer were generated in UCSF ChimeraX (v1.6.1). A heparan sulfate fragment was docked to the BA.2.86 and BA.2.86-T356K RBD using the Molecular operating environment (MOE) software (Version 2020.09). The docking was done with default parameters. Briefly, we used “General” mode to run docking. For Placement step, Triangle Matcher is selected for method, London dG is selected for Score and 30 is set for Poses. For Refinement step, Rigid Receptor is selected for method, GBVI/WSA dG is selected for Score and 5 is set for Poses.

**Infectivity assay**

Spike-pseudotyped VSV are prepared as described previously [5]. The spike genes (D614G, Delta, BA.1, BA.2, BA.5, BA.5-K356T, BA.2.75, BA.2.75-K356T, XBB.1.5, XBB.1.5-K356T, EG.5.1, BA.2.86, BA.2.86-T356K, JN.1) were optimized using mammalian codons and inserted into the pcDNA3.1 vector. Afterwards, the plasmids were transfected into 293T cells using Lipofectamine 3000 (Invitrogen). These cells were separately infected with G*ΔG-VSV pseudotyped virus (Kerafast) and virus-like particles (SC2-VLPs) pseudovirus. After incubation, the supernatant containing the pseudovirus was collected, filtered through a 0.45 μm filter membrane, and stored at -80℃ for future use.

We used HEK293T-hACE2 cells, Vero cells and Huh-7 cells as targets in infectivity assays. After quantification using RT-PCR, 100 μl aliquots of the diluted virus were introduced into individual wells of 96-well cell culture plates. Chemiluminescence monitoring was conducted following a 24-hour incubation period with a temperature of 37°C and a CO_2_ concentration of 5%. The supernatant for each sample was adjusted to a volume of 100 μl to ensure consistency. A mixture of luciferase substrate and cell lysis buffer (PerkinElmer, Fremont, CA) was prepared and added to each well at a volume of 100 μl. 150 μl of the resulting lysate was transferred to opaque 96-well plates after 2 min. 2 PerkinElmer Ensight luminometer was used to detect the luminescence signal, and the data was recorded in terms of relative luminescence unit (RLU) values. Each experimental group consisted of two replicates and the entire set of experiments was repeated three times. For infectivity assay related to heparin sulfate (HS), Virus-like particles (SC2-VLPs) were selected to infect HEK293T cells overexpressing ACE2 and Furin (293T-ACE2/Furin) treated with various concentrations of free heparin sulfate (HS).

**Western blot analysis**

Cell-cell fusion assays were described previously [6]. At 48 h of infection, cells and culture medium were collected. The culture media were centrifuged and the supernatants were collected. After that, an equal volume of clarified supernatants was mixed with 20% PEG6000 in PBS and centrifuged at 12,000g for 30 min at 4 °C, followed by pellet resuspension in 1× SDS sample buffer.

For cell lysates, the collected cells were washed and lysed in lysis buffer (Cell Signalling) and the lysates were diluted with 4 × sample buffer (Bio-Rad) and boiled for 10 min before analysed using western blotting. The following antibodies were used for protein detection: mouse anti-SARS-CoV-2 S1 antibodies (MAB105403, R&D systems), rabbit anti-SARS-CoV-2 S monoclonal antibodies (PA1-41165, Thermo Fisher Scientific), horseradish peroxidase (HRP)-conjugated anti-rabbit and anti-mouse IgG polyclonal antibodies (Cell Signalling). The ChemiDoc Touch Imaging System (Bio-Rad) was used to detected Chemiluminescence. The cleavage ratio of S1 or S2 to FL in virions was determined by densitometry using ImageJ software (NIH).

**Cell-cell fusion assay**

Cell-cell fusion assays were described previously [7]. In brief, HEK293T GFP11 and Vero-GFP1-10 cells were seeded at 80% confluence at a 1:1 ratio in 48-well plates the day before. Cells were co-transfected with 0.5 µg of spike expression plasmids. An Incucyte was used to measure cell–cell fusion and fusion was determined as the proportion of green area to total phase area. To measure cell surface spike expression, HEK293 cells were transfected with S expression plasmids and stained with rabbit anti-SARS-CoV-2 S S1/S2 polyclonal antibodies (Thermo Fisher Scientific, PA5-112048, 1:100). Negative control is normal rabbit IgG (SouthernBiotech, 0111-01, 1:100), and Secondary antibodies are APC-conjugated goat anti-rabbit IgG polyclonal antibodies (Jackson ImmunoResearch, 111-136-144, 1:50). The surface expression level of S proteins was analysed using FACS Canto II (BD Biosciences) and FlowJo v.10.7.1 (BD Biosciences).

**Antibody expression and purification**

SARS-CoV-2 RBD-specific mAbs were synthesized as described previously. Briefly, antibody heavy and light chain genes were synthesized by GenScript, inserted into pCMV3-CH, pCMV3-CL or pCMV3-CK vector plasmids by infusion (Vazyme), and co-transfected into Expi293F cells (Thermo Fisher) using polyethylenimine. Transfected cells were cultured at 36.5°C in 5% CO_2_ and 175 rpm for 6-10 days. Expression fluid was then collected and centrifuged, and the supernatants containing monoclonal antibodies were purified with Protein A magnetic beads (GenScript). Purified antibodies were verified by SDS-PAGE.

**Pseudovirus neutralization assay**

SARS-CoV-2 variants (BA.5, BA.2.75, BQ.1.1, XBB.1.5, XBB.1.5-K356T, EG.5.1, BA.2.86, BA.2.86-T356K, JN.1) spike-pseudotyped virus was constructed based on a vesicular stomatitis virus (VSV) pseudovirus packaging system, as described previously. Spike gene is inserted into pcDNA3.1 vectors. G*ΔG-VSV virus (VSV G pseudotyped virus, Kerafast) and spike plasmids were transfected to HEK293T cells (American Type Culture Collection [ATCC], CRL-3216). After culture, the pseudovirus in the supernatant was harvested, filtered, aliquoted, and frozen at −80°C for further use.

We used Huh-7 cells (Japanese Collection of Research Bioresources [JCRB], 0403) as targets in pseudovirus neutralization assays. Plasma samples or mAbs were serially diluted in culture media and mixed with pseudovirus, and incubated for 1 h in a 37°C incubator with 5% CO_2_. Digested Huh-7 cells were seeded in the antibody-virus mixture. After 1 day incubation, the supernatant was discarded. D-luciferin reagent (PerkinElmer, 6066769) was added into the plates and incubated in the dark for 2 min, and cell lysis was transferred to plates for detection. The luminescence values were measured by a microplate spectrophotometer (PerkinElmer, HH3400). IC50 values for mAbs and NT50 values for plasma were determined by fitting a logistic regression model.

**ADCC assays**

The full-length spike gene sequence of SARS-CoV-2 (GenBank: MN908947) was synthesized, with mutations for prefusion stabilization and modification on the furin cleavage site (Spike-6P2A or Spike-6P/GSAS). The Spike gene was inserted into the pLVX-puro vector. HEK293T cells (10 million) were transfected with 20.7 μg helper plasmid (pSPAX2), 13.8 μg VSV-G expression plasmid (pMD2.G) and 1 μg full-length spike expression vector (pLVX-puro) using the PEI ﻿transfection system (Yeasen, 40816ES03) to generate the lentivirus. Cell supernatant containing lentivirus was collected 48 hours after transfection, centrifuge at 500 g at 4 °C for 10 min and ﬁltered through a surfactant-free cellulose acetate 0.45 mm syringe ﬁlter. 5 ml lentivirus was used to infect 1 million low passage HEK293T cells. At 72 hours post infection, cells were stained with SA55-FITC for RBD labeling, single cell clone was sorted into a 96-well plate containing selective medium (DMEM+10 % FBS + 10 μg/ml puromycin + 1 % penicillin-streptomycin solution) using BD Aria II cell sorter in FITC channel. Single clone cell was expanded and tested for the Spike expression level via continuous puromycin selection and flow cytometry cell sortings. The clone with the highest Spike expression level (target cells) for amplification and used to evaluate the ADCC effect of antibodies.

The ADCC effector cells (Human CD16a Jurkat reporter cells) are gift from Youchun Wang, which were engineered to express both the NFAT response element driving luciferase expressing systems and human CD16a receptor with 158V mutation for higher affinity to IgG1 and IgG3 isotypes. Similarly, we used flow cytometry to analyze the expression of human CD16 on the effector cells. Cells were incubated with BD Pharmingen PE Mouse Anti-Human CD16 and subjected for FACS analyses using BD Aria II cell sorter.

To detect antibodies’ potency to mediate ADCC, mAbs were pre-diluted in a reaction medium of 1640 medium (HyClone) containing 10% FBS (Gibco) and 1% penicillin-streptomycin solution. Add serial dilutions of antibodies in 10 μl to wells. Meanwhile, add 10 μl of reaction medium to the unstimulated control wells. Digest target cells and plate the target cells at a density of 1.67 ×10^6^ cells/ml in 384-well culture plates in 10 μl of reaction medium, then aliquots of pre-diluted antibodies were incubated with target cells in 384-well culture plates for 10 minutes at 37 ℃ in the 5% CO_2_ incubator. Subsequently, add the effector cells (Human CD16a Jurkat reporter cells) at a density of 1.67 ×10^6^ cells/ml in 10 μl of reaction medium to the culture wells. The mixtures were further incubated at 37℃ in the 5% CO_2_ incubator for 18 hours. Finally, add 30 μl reagent of Stable-Lite Luciferase Assay System (Vazyme, DD1202) to each well and incubate in the dark for 2 minutes. The chemiluminescence signals were collected by PerkinElmer Ensight. The ADCC luciferase (luc) fold induction was calculated by the fold change of the relative light unit (RLU) values of test wells to control blank wells. The area under curve (AUC, log-concentration v.s. log-fold-change) values are calculated and used as a metric to evaluate the capability of each antibody to mediate ADCC. ADCC assays for all mAbs were conducted in two replicates.

**Protein vaccine preparation and mouse immunization**

The spike proteins, including BA.5, EG.5.1, XBB.1.5, BA.2.86 and BA.2.86-T356K were used for mouse immunization. All of these proteins were purified as previously described.

Animal experiments were carried out under study protocols approved by Rodent Experimental Animal Management Committee of Institute of Biophysics, Chinese Academy of Sciences (SYXK2023300) and Animal Welfare Ethics Committee of HFK Biologics (HFK-AP-20210930). Six- to eight-week-old female BALB/c mice were used for experiments. The mice were kept under a 12-hour light and 12-hour dark cycle, with room temperatures maintained between 20 °C and 26 °C. The humidity levels in the housing area ranged from 30% to 70%. Mice were immunized according to schemes in Fig. 7. Briefly, two cohorts of BALB/c mice were established to evaluate the immunogenic of various SARS-CoV-2 variants. For the first cohort, mice had a pre-existing immune background and received 0.3 μg SARS-CoV-2 WT inactivated vaccine (CoronaVac, against SARS-CoV-2 wild-type) as the primary vaccination at six- to eight weeks age, followed by a booster dose after 14 days. When these mice reached 5.5 months of age, they were given an additional boost using the omicron (BA.5) inactivated vaccine (against SARS-CoV-2 BA.5 variant) with a dose of 0.3 μg as well. For the last dose of this cohort, the mice were intramuscularly administered 10 μg, 1 mg/ml of spike protein from 5 different variants (BA.5, XBB.1.5, EG.5.1, BA.2.86 and BA.2.86-T356K) as immunogen. For the other cohort, mice did not undergo any form of immunization. They were intramuscularly administered 2 doses of 10 μg, 1 mg/ml of 5 types of spike proteins as described before. The interval between the two injections is 14 days. The adjuvants for inactivated vaccines and proteins are Al+CpG. Blood samples were collected on the 14th day post-immunization, and serum was obtained through centrifugation.

**REFERENCES**

1. Alachiotis N, Stamatakis A and Pavlidis P. OmegaPlus: a scalable tool for rapid detection of selective sweeps in whole-genome datasets. *Bioinformatics* 2012; **28**: 2274-2275.

2. Alachiotis N and Pavlidis P. RAiSD detects positive selection based on multiple signatures of a selective sweep and SNP vectors. *Commun Biol* 2018; **1**: 79.

3. Li H. Minimap2: pairwise alignment for nucleotide sequences. *Bioinformatics* 2018; **34**: 3094-3100.

4. Katoh K and Standley DM. MAFFT Multiple Sequence Alignment Software Version 7: Improvements in Performance and Usability. *Molecular Biology and Evolution* 2013; **30**: 772-780.

5. Li Q, Wu J and Nie J *et al*. The Impact of Mutations in SARS-CoV-2 Spike on Viral Infectivity and Antigenicity. *Cell* 2020; **182**: 1284-1294.e1289.

6. Meng B, Datir R and Choi J *et al.* SARS-CoV-2 spike N-terminal domain modulates TMPRSS2-dependent viral entry and fusogenicity. *Cell Reports* 2022; **40**: 111220.

7. Meng B, Abdullahi A and Ferreira I *et al*. Altered TMPRSS2 usage by SARS-CoV-2 Omicron impacts infectivity and fusogenicity. *Nature* 2022; **603**: 706-714.

**
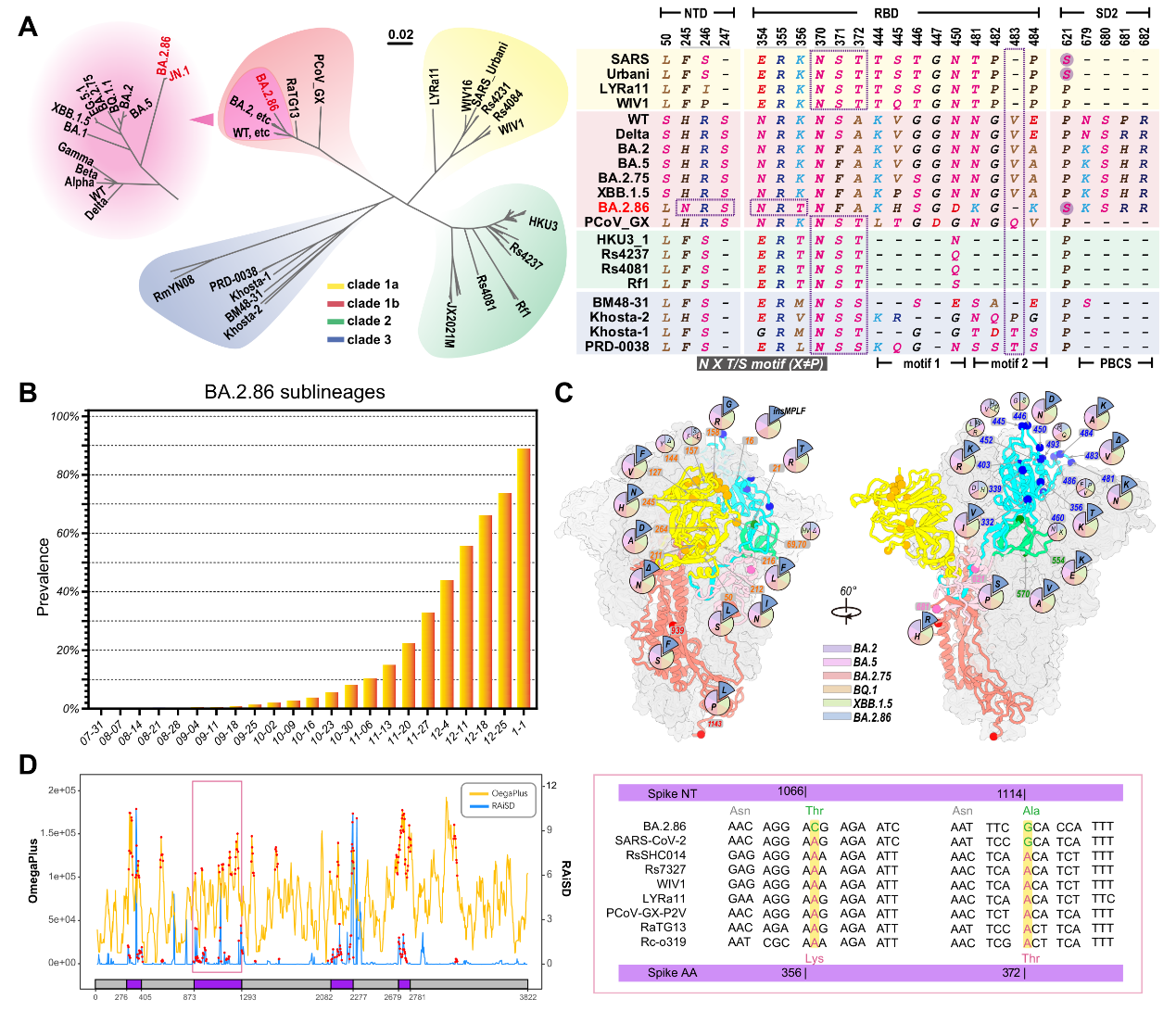
Supplementary Figure S1. Selective advantages in the Spike gene.** (**A**) Phylogenetic tree of sarbecoviruses based on S sequences and sequence features of S protein of selected variants from four clades. At the left panel, four clades are shadowed in yellow (clade 1a), red (clade 1b), green (clade 2) and blue (clade 3), respectively. For clade 1b, lineages of SARS-CoV-2 are zoomed in. Some typical variants are labeled and BA.2.86 and JN.1 are highlighted. At the right panel, motifs satisfying N-glycosylation (NXS/T, X≠P) are circled and residue 483 and 621 on S are highlighted. (**B**) Bar chart of the relative prevalence of BA.2.86 sublineages as of the first week of 2024. (**C**) Locations and residue diversities of mutations carried by BA.2.86 spike. Only residues on S differing from BA.2 are selected. Mutations unique to BA.2.86 are highlighted by larger detached sector diagram. For color scheme, NTD, RBD, SD1, SD2 and S2 are colored in yellow, cyan, green, pink and light red. The CA atoms of mutated residues are shown as spheres. (**D**) Selective sweep regions (shown as purple blocks) identified in SARS-CoV-2 genomes using OmegaPlus (yellow lines) and RAiSD (blue lines). Important non-synonymous differences are highlighted at the right panel.


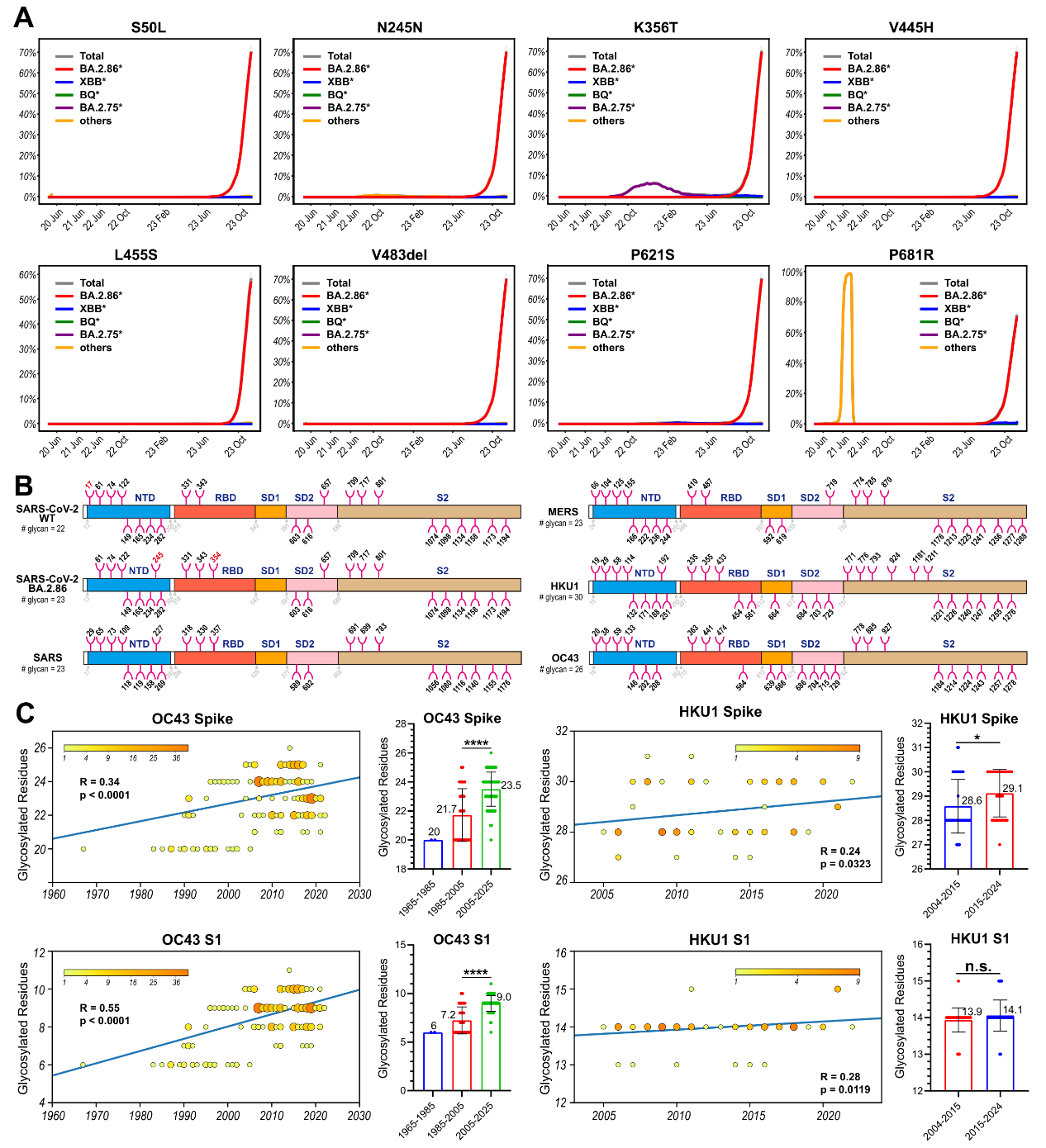
**Supplementary Figure S2. Proportion of typical mutations carried by BA.2.86 spike and distribution of coronavirus glycosylation residues on spike.** (**A**) Proportion of SARS-CoV-2 spike residue mutations S50L, H245N, K356T, L455S, V455H, V483del, P621S and P681R from January 2020 to January 2024. (**B**) A schematic diagram of sequence glycosylation site on SARS-CoV-2 WT, SARS-CoV-2 BA.2.86, SARS, MERS, HKU1 and OC43 spike predicted by “NXS/T” rule. (**C**) Correlation between the number of glycosylated residues on Spike and S1 over time for OC43 and HKU1.

**
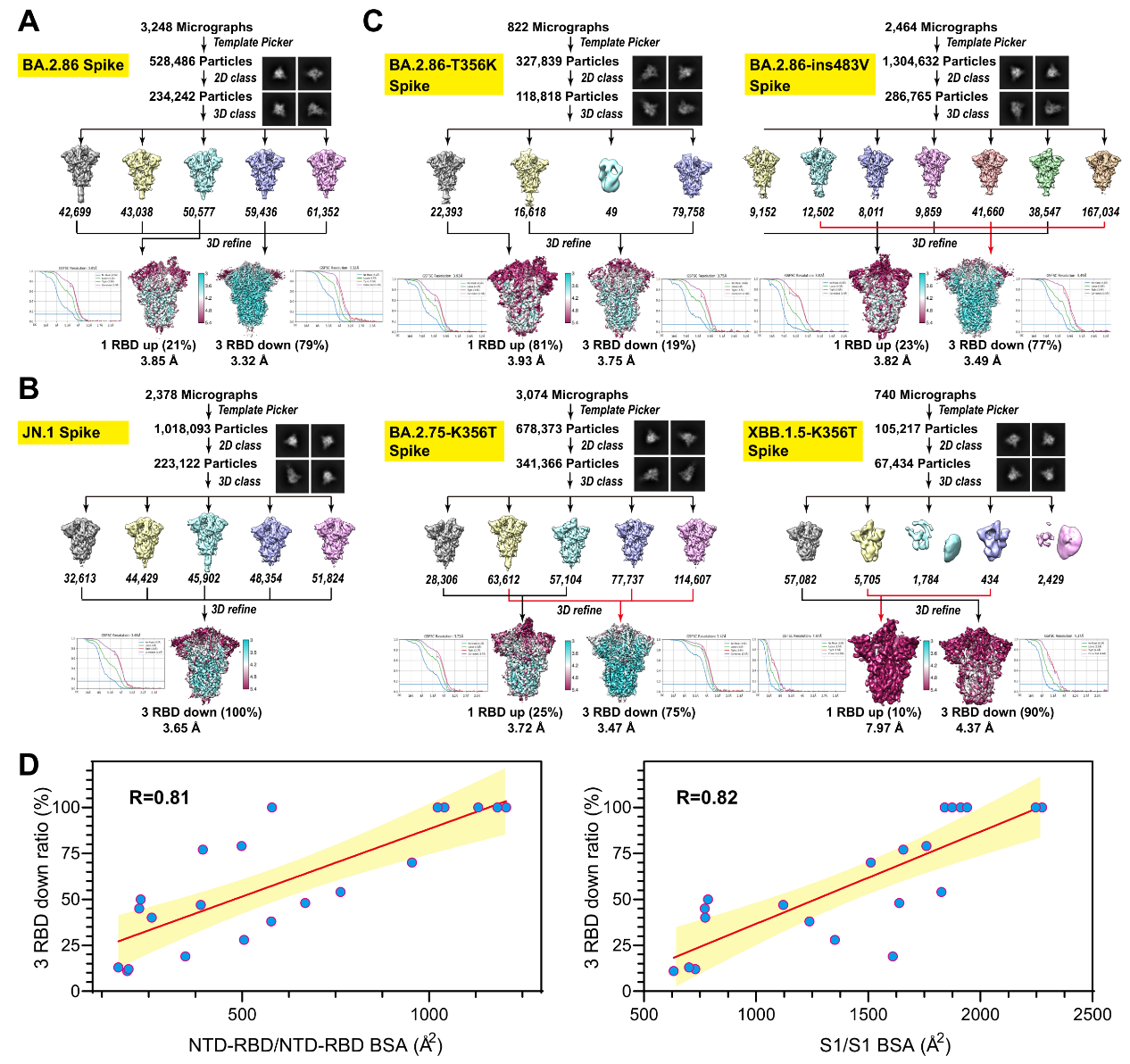
Supplementary Figure S3. Cryo-EM structures of S-trimer of 6 SARS-CoV-2 variants and linear regression analysis of “RBD down” conformation ratio to buried surface areas (BSA).** Flow charts, FSC curves and local resolutions of Cryo-EM structure BA.2.86 S-trimer (**A**), JN.1 S-trimer (**B**), BA.2.86-T356K S-trimer, BA.2.86-ins483V S-trimer, BA.2.75-K356T S-trimer and XBB.1.5-K356T S-trimer (**C**). (**D**) Correlation plots of ratio of spike with “3 RBD down” conformation with NTD-RBD/NTD-RBD BSA and S1/S1 BSA.

**
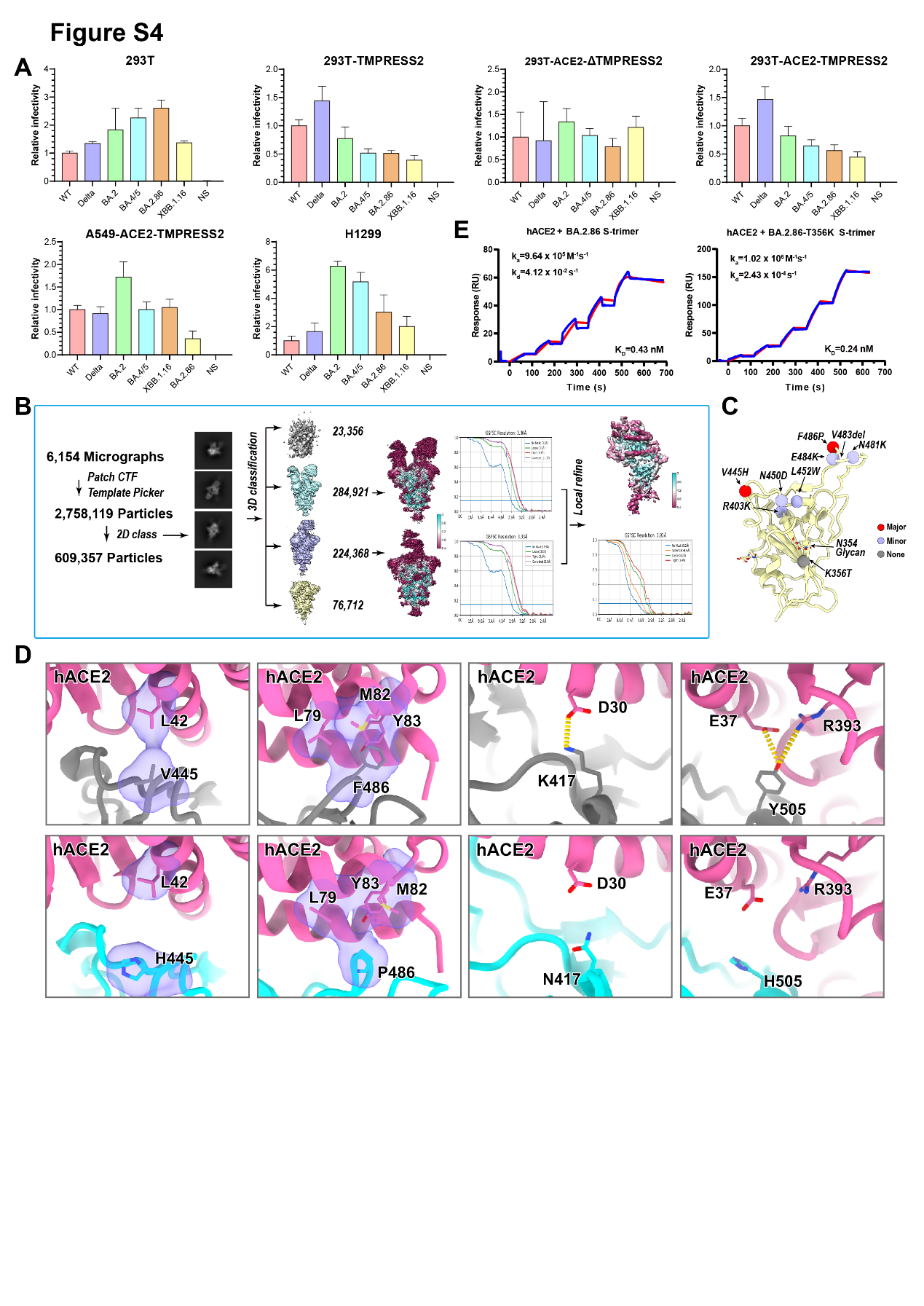
Supplementary Figure S4. Relative infectivity of SARS-CoV-2 variants in 7 cell lines, cryo-EM structure of BA.2.86 S-trimer with hACE2 and interface between hACE2 and RBD.** (**A**) Normalized SARS-CoV-2 variants pseudovirues entry in HEK293T cells (293T), HEK293T TMPRSS2-overexpressing cells (293T-TMPRSS2), HEK293T cells overexpressing ACE2 and deleted for TMPRSS2 (293T-ACE2-ΔTMPRSS2), HEK293T cells overexpressing ACE2 and TMPRSS2 (293T-ACE2-TMPRSS2), A549 cells overexpressing ACE2 and TMPRSS2 (A549-ACE2-TMPRSS2) and H1299 lung cells. Error bars represent the mean ± SD of three replicates. (**B**) Flow chart, FSC curve and local resolution of Cryo-EM structure of BA.2.86 S-trimer in complex with hACE2. (**C**) Locations of residues on BA.2.86 RBD that play a major (red), minor (light purple) and no (gray) role in binding affinity to hACE2. (**D**) Structure interpretation of mutation H445V, P486F, N417K and H505Y on BA.2.86 RBD increasing the binding affinity to hACE2. Residues associated with affinity change are shown as sticks. Hydrophobic network is highlighted in light purple and hydrogen bonds are presented as yellow dashed lines. Oxygen atoms are colored in red and nitrogen atoms are colored in blue. (**E**) The impact of glycosylation at position 354 of BA.2.86 S-trimer on the binding affinity to hACE2 was assessed by SPR.

**
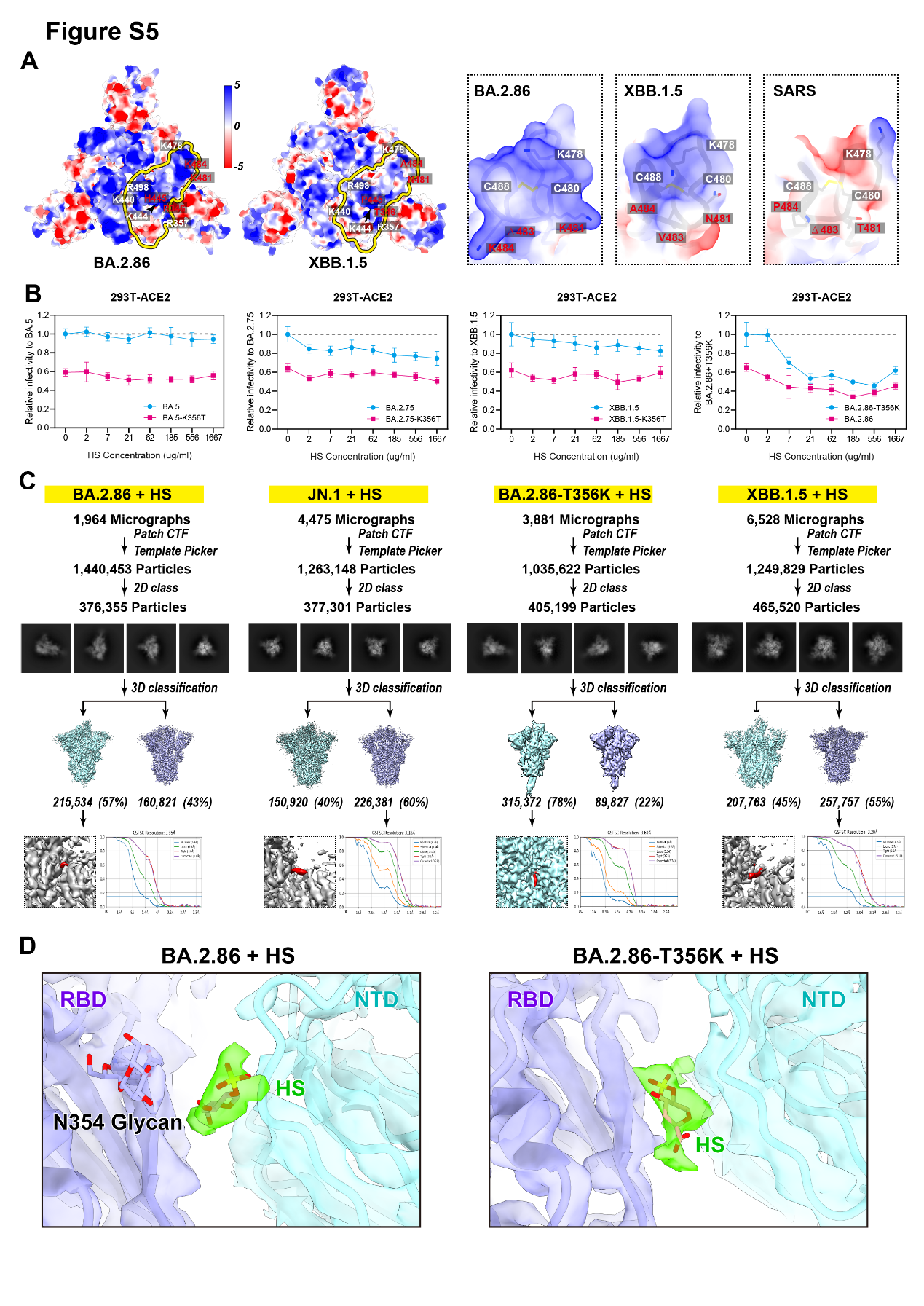
Supplementary Figure S5. Electrostatic surface of S1 subunit, infectivity with HS treatment and Cryo-EM structure of S-trimer bound to HS.** (**A**) Electrostatic surface of BA.2.86 (left) and XBB.1.5 (right) S1 subunit. Yellow circle marked a single RBD. Key residues are labeled and diverse residues are highlighted in red. Electrostatic surface of motif 2 on RBD of BA.2.86, XBB.1.5, and SARS are zoomed in. (**B**) BA.5, BA.2.75, XBB.1.5, BA.2.86-T356K and their corresponding K356T mutant VSV-based pseudoviruses entry HEK293T cells overexpressing ACE2 (293T-ACE2) treated with various concentrations of free HS. Error bars represent the mean ± SD of three replicates. (**C**) Flow charts and FSC curves of Cryo-EM structure of S-trimer of BA.2.86, JN.1, BA.2.86-T356K and XBB.1.5 in complex with HS. The density belonging to HS are highlighted in red. (**D**) Cryo-EM density maps of BA.2.86 Spike in complex with HS (left) and BA.2.86-T356K Spike in complex with HS (right). RBD, NTD and HS are colored in blue, cyan and green, respectively.

**
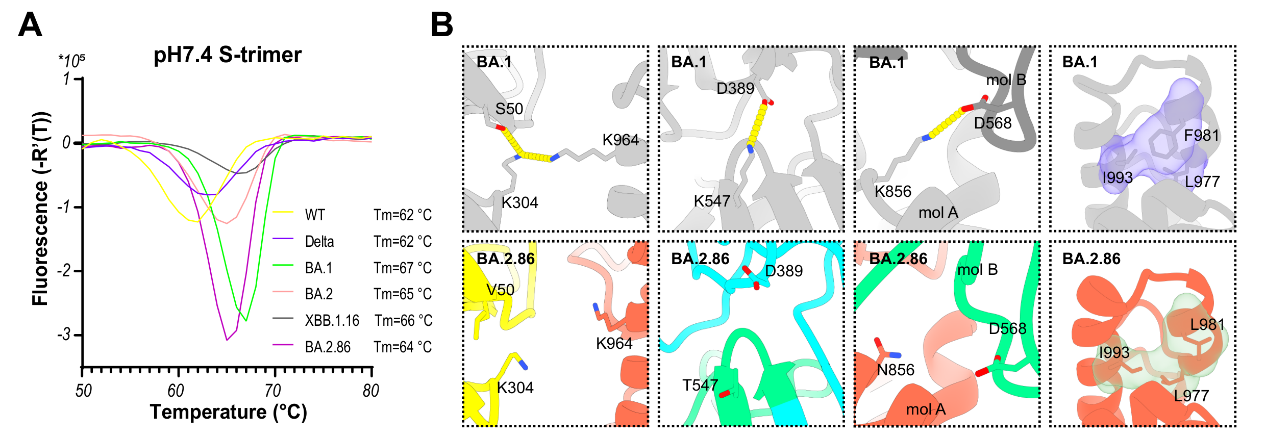
Supplementary Figure S6. Thermal stability analysis of S-trimer of SARS-CoV-2 variants.** (**A**) Thermal stability of WT, Delta, BA.1, BA.2, XBB.1.16 and BA.2.86 S-trimer measured by ThermoFluor Assay at neutral pH. (**B**) Zoomed-in view of the inter- and intra-subunits of S-trimer interaction details of BA.1 (top) and BA.2.86 (bottom). The residues involved in the interactions are shown as sticks. The hydrogen bonds are shown as yellow dashed lines and hydrophobic network is highlighted in light purple and light green. Different monomers on the same S-trimer are defined as mol A and mol B, respectively.


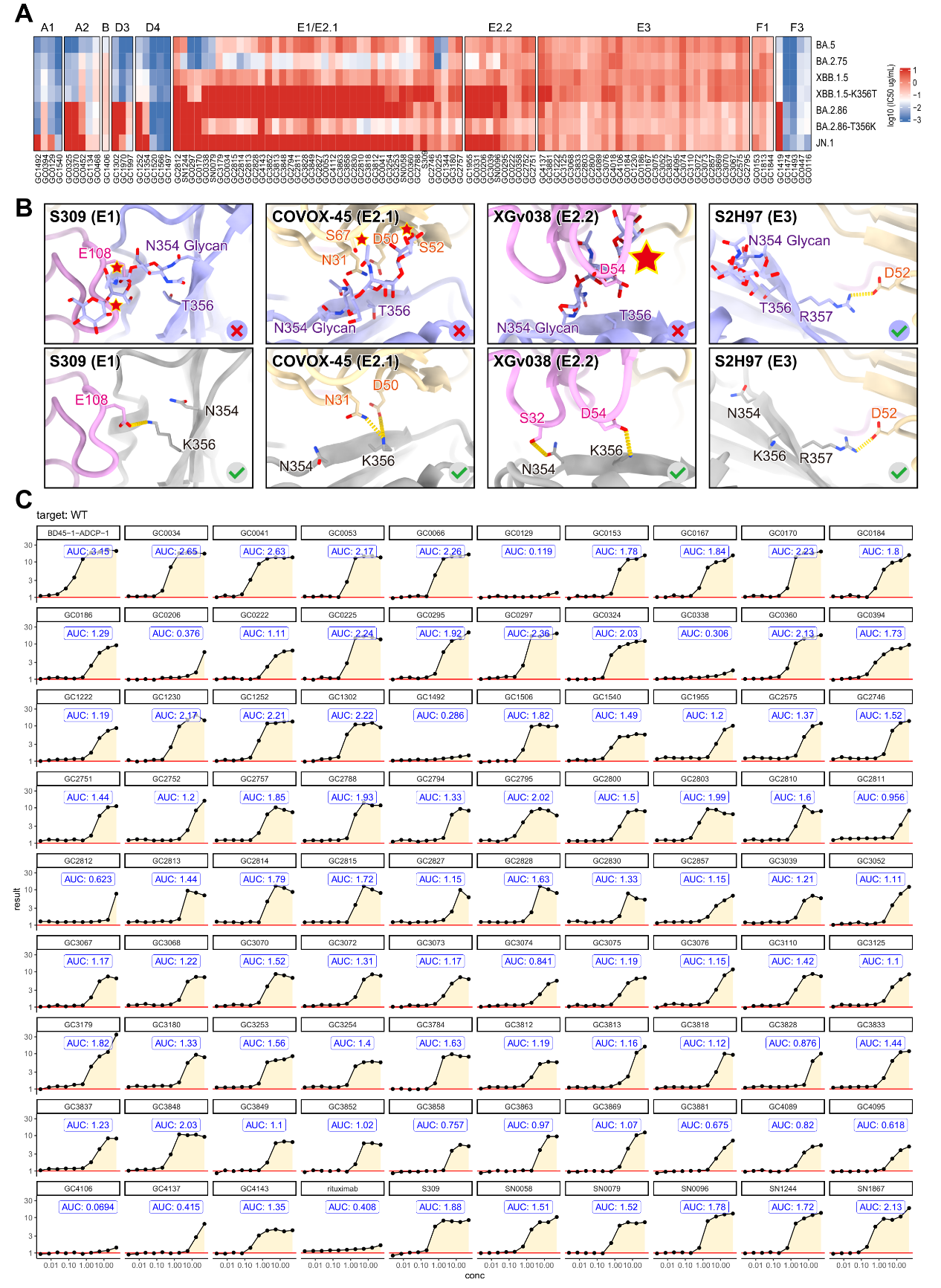
**Supplementary Figure S7. Pseudovirus neutralization assay, structural interpretation of the evasion of antibodies and ADCC assay of antibodies.** (**A**) Heatmap of log10 IC50 of antibodies from A1, A2, B, D3, D4, E1, E2.1, E2.2, E3, F1 and F3 epitope groups against BA.5, BA.2.75, XBB.1.5, XBB.1.5-K356T, BA.2.86, BA.2.86-T356K and JN.1 pseudovirus. (**B**) Cartoon representation of RBD with N354 glycosylation (top) and without N354 glycosylation (bottom) in complex with antibodies S309 (E1), COVOX-45 (E2.1), XGv038 (E2.2) and S2H97 (E3). The key residues of RBDs and antibodies participating interactions are shown as sticks. Atom clashes are shown as red star. The hydrogen bonds are shown as yellow dashed lines. For color scheme, RBD with and without N354 glycosylation are colored in light gray and gray, respectively. Light and heavy chain of antibodies are colored in light yellow and light pink, respectively. (**C**) The raw data of antibodies ADCC.


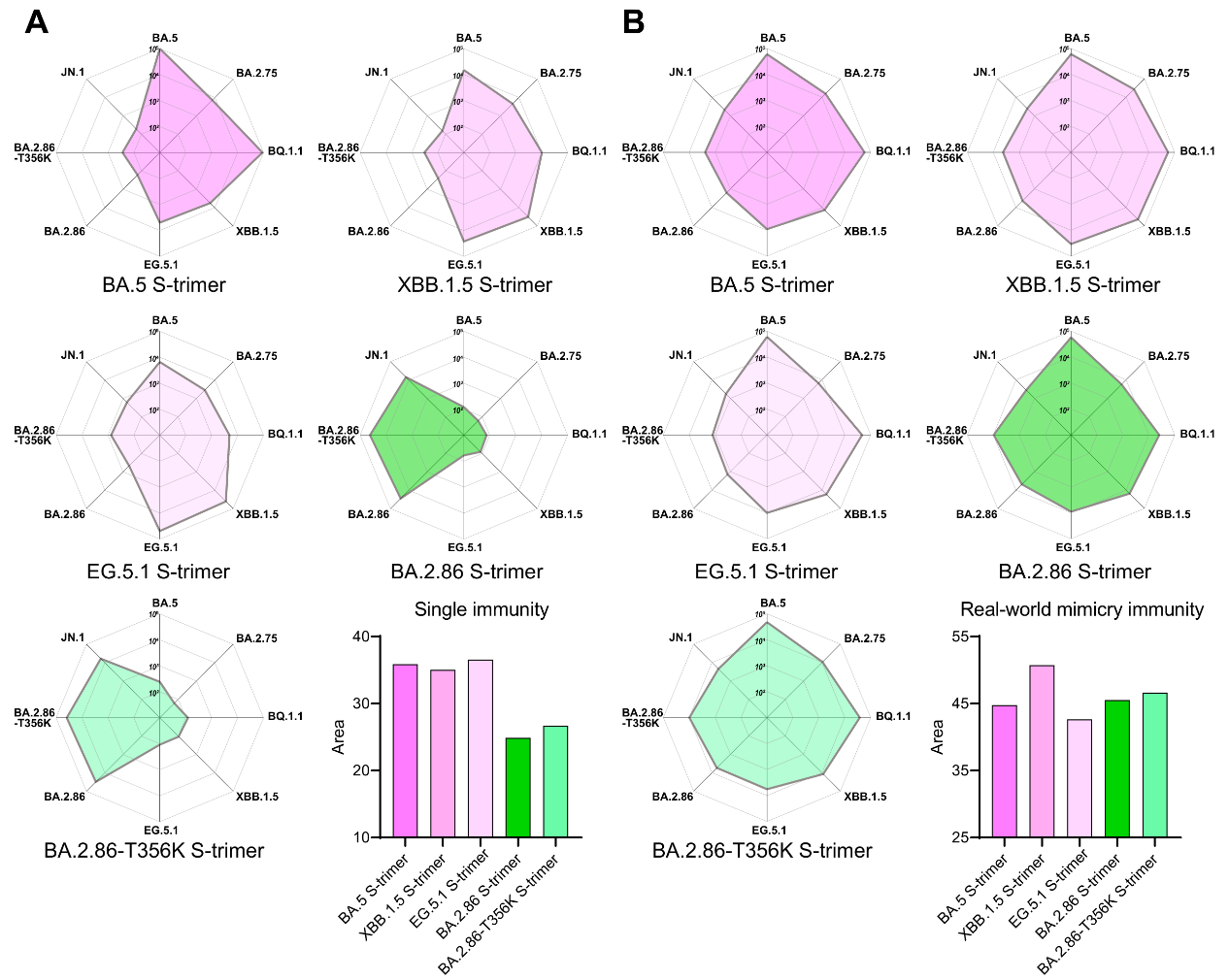
**Supplementary Figure S8. Immunogenicity of BA.2.86 relative to BA.5, XBB.1.5, EG.5.1, and BA.2.86-T356K S-trimer.** Radar plot of immunogenicity of BA.5, XBB.1.5, EG.5.1, BA.2.86 and BA.2.86-T356K S-trimer under single immunity background (**A**) and real-world mimicry immunity background (**B**). The serum neutralizing titers (NT_50_) against 8 pseudovirus (BA.5, BA.2.75, BQ.1.1, XBB.1.5, EG.5.1, BA.2.86 and BA.2.86-T356K) are log10-scaled to generate the radar plot and to calculate the area of the radar plot, which is displayed by bar chart. Color schemes are consistent with Figure 6.

**Table S1. Cryo-EM data collection, processing, and validation statistics**

| **Data collection** |  |  |  |
| --- | --- | --- | --- |
| Complex | BA.2.86 Spike (3 RBD down) | BA.2.86 Spike (1 RBD up) | JN.1 Spike (3 RBD down) |
| Microscope | FEI Talos Arctica | FEI Talos Arctica | FEI Talos Arctica |
| Camera | Gatan K2 | Gatan K2 | Gatan K2 |
| Voltage (kV) | 200 | 200 | 200 |
| Total dose (e^-^/A^2^) | 60 | 60 | 60 |
| Micrographs (total) | 3,248 | 3,248 | 2,378 |
| Micrographs (used) | 2,342 | 2,342 | 2,103 |
| Particles selected | 528,486 | 528,486 | 1,018,093 |
| Particles included in final reconstruction | 174,806 | 59,436 | 223,122 |
| sampling, Å per pixel | 1 | 1 | 1 |
| Defocus range (μm) | -1.2 ~ -2.0 | -1.2 ~ -2.0 | -1.2 ~ -2.0 |
| Symmetry | C3 | C1 | C3 |
| Resolution (Å) (FSC=0.143 criterion) | 3.32 | 3.85 | 3.65 |
| **Model refinement** |  |  |  |
| Ramachandran statistics (%) |  |  |  |
| Most favored | 91.86 | 91.42 | 91.58 |
| Allowed | 7.95 | 8.23 | 8.29 |
| Outliers | 0.19 | 0.35 | 0.13 |
| Bonds (RMSD) |  |  |  |
| Bond lengths (Å) | 0.011 | 0.006 | 0.009 |
| Bond angles (°) | 0.902 | 0.834 | 0.949 |
| MolProbity score | 2.2 | 2.27 | 2.2 |
| Clash score | 15.95 | 18.49 | 15.68 |
| Rama-Z |  |  |  |
| Rotamer outliers (%) | 0.79 | 1.01 | 0.32 |
| Cβ outliers (%) | 0 | 0 | 0.07 |
| **Deposit** |  |  |  |
| PDB | 8WHV | 8WHW | 8X4H |
| EMDB | EMD-37549 | EMD-37550 | EMD-38049 |

|  |  |  |  |  |
| --- | --- | --- | --- | --- |
| BA.2.86-T356K Spike (3 RBD down) | BA.2.86-T356K Spike (1 RBD up) | BA.2.86-ins483V Spike (3 RBD down) | BA.2.86-ins483V Spike (1 RBD up) | BA.2.75-K356T Spike (3 RBD down) |
| FEI TITAN | FEI TITAN | FEI TITAN | FEI TITAN | FEI Talos Arctica |
| Gantan K2 | Gantan K2 | Gantan K2 | Gantan K2 | Gatan K2 |
| 300 | 300 | 300 | 300 | 200 |
| 60 | 60 | 60 | 60 | 60 |
| 822 | 822 | 2,464 | 2,464 | 3,074 |
| 822 | 822 | 2,464 | 2,464 | 3,074 |
| 327,839 | 327,839 | 1,304,632 | 1,304,632 | 678,373 |
| 96,376 | 22,393 | 221,196 | 65,569 | 163,826 |
| 1.04 | 1.04 | 1.04 | 1.04 | 1 |
| -1.2 ~ -2.0 | -1.2 ~ -2.0 | -1.2 ~ -2.0 | -1.2 ~ -2.0 | -1.2 ~ -2.0 |
| C3 | C1 | C3 | C1 | C3 |
| 3.75 | 3.93 | 3.49 | 3.82 | 3.47 |
|  |  |  |  |  |
|  |  |  |  |  |
| 92.5 | 92.72 | 92.25 | 92 | 90.94 |
| 7.38 | 7.19 | 7.65 | 7.91 | 8.69 |
| 0.13 | 0.09 | 0.09 | 0.09 | 0.37 |
|  |  |  |  |  |
| 0.004 | 0.005 | 0.004 | 0.004 | 0.012 |
| 0.739 | 0.756 | 0.74 | 0.721 | 0.999 |
| 2.11 | 2.16 | 2.07 | 2.19 | 2.19 |
| 13.42 | 15.83 | 12.05 | 15.82 | 14.61 |
|  |  |  |  |  |
| 0.36 | 0.36 | 0.47 | 0.36 | 0.82 |
| 0 | 0 | 0 | 0 | 0.03 |
|  |  |  |  |  |
| 8X55 | 8X56 | 8X4Z | 8X50 | 8X5Q |
| EMD-38063 | EMD-38064 | EMD-38056 | EMD-38057 | EMD-38072 |

|  |  |  |  |  |
| --- | --- | --- | --- | --- |
| BA.2.75-K356T Spike (1 RBD up) | XBB.1.5-K356T Spike (3 RBD down) | XBB.1.5-K356T Spike (1 RBD up) | BA.2.86 Spike+hACE2 (bound to 1 ACE2) | BA.2.86 Spike+hACE2 (bound to 2 ACE2) |
| FEI Talos Arctica | FEI Talos Arctica | FEI Talos Arctica | FEI TITAN | FEI TITAN |
| Gatan K2 | Gatan K2 | Gatan K2 | Gatan K3 | Gatan K3 |
| 200 | 200 | 200 | 300 | 300 |
| 60 | 60 | 60 | 60 | 60 |
| 3,074 | 740 | 740 | 6,154 | 6,154 |
| 3,074 | 740 | 740 | 6,154 | 6,154 |
| 678,373 | 105,217 | 105,217 | 2,758,119 | 2,758,119 |
| 93,276 | 57,082 | 6,139 | 224,368 | 284,921 |
| 1 | 1 | 1 | 1.07 | 1.07 |
| -1.2 ~ -2.0 | -1.2 ~ -2.0 | -1.2 ~ -2.0 | -1.2 ~ -2.0 | -1.2 ~ -2.0 |
| C1 | C3 | C1 | C1 | C1 |
| 3.72 | 4.37 | 7.97 | 3.33 | 3.3 |
|  |  |  |  |  |
|  |  |  |  |  |
| 90.38 | -- | -- | 92.26 | 93.07 |
| 9.21 | -- | -- | 7.52 | 6.88 |
| 0.4 | -- | -- | 0.21 | 0.05 |
|  |  |  |  |  |
| 0.005 | -- | -- | 0.007 | 0.014 |
| 0.911 | -- | -- | 0.897 | 1.202 |
| 2.27 | -- | -- | 2.1 | 2.21 |
| 17.2 | -- | -- | 12.83 | 18.6 |
|  |  |  |  |  |
| 0.53 | -- | -- | 0.88 | 0.1 |
| 0 | -- | -- | 0.06 | 0.02 |
|  |  |  |  |  |
| 8X5R | -- | -- | 8WHS | 8WHU |
| EMD-38073 | EMD-38701 | EMD-38700 | EMD-37546 | EMD-37548 |

|  |  |  |  |  |
| --- | --- | --- | --- | --- |
| BA.2.86 Spike+hACE2 (local refinement) | BA.2.86 Spike + HS | JN.1 Spike + HS | BA.2.86-T356K Spike + HS (local refine) | XBB.1.5 Spike + HS |
| FEI TITAN | FEI TITAN | FEI Krios G4 | FEI TITAN | FEI Krios G4 |
| Gatan K3 | Gatan K3 | Falcon 4 | Gatan K2 | Falcon 4 |
| 300 | 300 | 300 | 300 | 300 |
| 60 | 60 | 60 | 60 | 60 |
| 6,154 | 1,954 | 4,475 | 3,881 | 6,528 |
| 6,154 | 1,964 | 4,475 | 3,881 | 6,528 |
| 2,758,119 | 1,440,453 | 1,263,148 | 1,035,622 | 1,249,829 |
| 157,447 | 215,534 | 301,841 | 315,372 | 257,757 |
| 1.07 | 1.07 | 1.036 | 1.04 | 1.036 |
| -1.2 ~ -2.0 | -1.2 ~ -2.0 | -1.2 ~ -2.0 | -1.2 ~ -2.0 | -1.2 ~ -2.0 |
| C1 | C1 | C1 | C1 | C3 |
| 3.93 | 3.85 | 3.18 | 3.69 | 3.2 |
|  |  |  |  |  |
|  |  |  |  |  |
| 95.81 | 91.92 | 91.26 | 88.12 | 88.63 |
| 4.07 | 7.58 | 8.1 | 11.16 | 11.14 |
| 0.13 | 0.51 | 0.63 | 0.71 | 0.23 |
|  |  |  |  |  |
| 0.005 | 0.01 | 0.008 | 0.005 | 0.011 |
| 1.058 | 0.9 | 0.85 | 0.806 | 1.124 |
| 1.93 | 2.45 | 2.13 | 2.2 | 2.43 |
| 13.69 | 16.5 | 12.7 | 12.28 | 22.7 |
|  |  |  |  |  |
| 0.14 | 1.05 | 0.47 | 0.26 | 0.04 |
| 0 | 0 | 5.4 | 0 | 0.1 |
|  |  |  |  |  |
| 8WHZ | 8XUR | 8XUS | 8XUU | 8XUT |
| EMD-37553 | EMD-38681 | EMD-38682 | EMD-38684 | EMD-38683 |
